# A Systematic Review of the Cardiometabolic Benefits of Plant Products Containing Mixed Phenolics and Polyphenols in Postmenopausal Women: Insufficient Evidence for Recommendations to This Specific Population

**DOI:** 10.3390/nu13124276

**Published:** 2021-11-27

**Authors:** Lorena Sánchez-Martínez, María-Jesús Periago, Javier García-Alonso, María-Teresa García-Conesa, Rocío González-Barrio

**Affiliations:** 1 Department of Food Technology, Food Science and Nutrition, Faculty of Veterinary Sciences, Regional Campus of International Excellence ‘Campus Mare Nostrum’, Biomedical Research Institute of Murcia (IMIB-Arrixaca-UMU), University Clinical Hospital ‘Virgen de la Arrixaca’, Universidad de Murcia, Campus de Espinardo, 30100 Murcia, Spain; lorena.sanchez14@um.es (L.S.-M.); mjperi@um.es (M.-J.P.); fjgarcia@um.es (J.G.-A.); 2 Research Group on Quality, Safety and Bioactivity of Plant Foods, Centro de Edafología y Biología Aplicada del Segura-Consejo Superior de Investigaciones Científicas (CEBAS-CSIC), Campus de Espinardo, P.O. Box 164, 30100 Murcia, Spain

**Keywords:** antioxidants, HOMA-IR, systolic blood pressure, diastolic blood pressure, TNF-α, CRP, IL-6, endothelial cell adhesion biomarkers, oxidative stress biomarkers, (poly)phenols

## Abstract

Menopause is characterized by endocrine and physiological changes and is often accompanied by increased body weight and cholesterol, glucose intolerance, and/or hypertension. These alterations are associated with increased risk for cardiovascular diseases (CVDs) and Type II diabetes mellitus (T2DM) that may be moderate by dietary plant phenolic compounds. In this review, we examine the current evidence of the impact of a variety of plant products (foods, extracts, beverages) rich in a mixture of phenolics and polyphenols on: (i) glucose and insulin levels; (ii) lipid profile; (iii) blood pressure; and (iv) biomarkers of inflammation and oxidative stress in postmenopausal women. We critically evaluate both the results of a range of intervention studies conducted in this specific subpopulation and the level of evidence supporting the benefits of consuming those products after the menopause. Overall, the current available evidence does not allow for specific dietary recommendations of these plant products rich in phenolics and polyphenols in this high-risk subpopulation. Our data show rather variable and small effects of the different products examined on the cardiometabolic biomarkers and further support the need to: (1) improve the quality of the study designs and data reporting; and (2) understand the variability in the response of the different biomarkers and establish clear differences between healthy and cardiometabolic disease levels.

## 1. Introduction

Cardiovascular diseases (CVDs) remain the leading cause of death worldwide [1], and have been described as the main cause of morbidity and mortality in women attributable to specific risk factors such as menopause. Menopause is a stage characterized by the sudden decrease in estrogens linked to the absence of amenorrhea for at least 12 months [2]. Estrogens are sex hormones that participate in the reproductive processes but also intervene in certain metabolic pathways such as lipids metabolism and the distribution of body fat in the body [3]. As a result of the fall of estrogen production, postmenopausal women experience a number of alterations of the energy homeostasis that are accompanied by an increase in body weight and a tendency to obesity and visceral fat deposition, as well as variations in the levels of total cholesterol (T-C), low-density lipoprotein-cholesterol (LDL-C), high-density lipoprotein-cholesterol (HDL-C), and triglycerides (TGs), increasing the risk for cardiometabolic diseases [4,5]. In addition, menopause contributes to disorders of the glucose metabolism increasing insulin resistance (IR) that can predispose one to the development of type 2 diabetes mellitus (T2DM) [6].

Metabolic syndrome (MetS) is defined as a cluster of some cardiometabolic alterations (i.e., weight excess, reduced HDL-C, and elevated levels of TGs, glucose and/or high blood pressure) [7]. MetS can also be found in association with a low-grade inflammation. Due to the accumulation of intracellular fat and the oxidation of fatty acids in the adipocytes, a lipotoxic reaction develops with the formation of free radicals, which triggers an inflammatory response and the increase of pro-inflammatory cytokines such as tumor necrosis factor-alpha (TNF-α), interleukin-6 (IL-6) and/or interleukin-1 (IL-1), C reactive protein (CRP), and the decrease in the secretion of the anti-inflammatory adipokine, adiponectin [8]. Often, postmenopausal women present a combination of two or three of these alterations and a higher prevalence of MetS compared to similar aged pre-menopausal women [9] raising their risk of CVDs and T2DM.

At present, hormone replacement therapy (HRT) is the most effective strategy for the management of menopause. However, it is associated with an increased risk for CVDs and breast cancer [10]. Apart from some pharmacological treatments (i.e., statins, antidiabetic drugs), general dietary and exercise guidelines can help to diminish the cardiometabolic risk factors associated with menopause. However, new therapies and strategies to reduce or moderate the CVDs and associated metabolic risk factors in postmenopausal women using natural compounds with little or no adverse effects are desirable.

For several decades now, plant-based diets rich in fruits and vegetables have been considered beneficial against the development of CVDs [11]. Among other dietary components (fiber, vitamins, minerals), plant foods are rich in secondary metabolites with a structure formed by an aromatic ring and one or more hydroxyl substituents. These compounds are generally involved in plant defense against abiotic and biotic stress [12]. They are classified as flavonoids (i.e., flavonols, flavones, isoflavones, flavanones, anthocyanidins, flavan-3-ols, and dihydrochalcones) and nonflavonoids (including phenolic acids, lignans, hydrolyzable tannins and stilbenes) depending on their chemical structure [13]. The terminology used to refer to all of these compounds is often field-specific and may lead to misunderstandings and problems with literature searches and systematic reviews. Recently, the nomenclature has been standardized and clarified with specific definitions for each term. In particular, the term (poly)phenols refers to a mixture containing a combination of phenolics (one phenol group only) and polyphenols (more than one phenol group) [14]. Many of these plant-derived compounds are recognized as both bioactive compounds with general antioxidant and anti-inflammatory activity, and as potent vasodilators and stimulators of the immune response with the capacity to modulate a range of cardiovascular and metabolic risk factors [15,16]. An increasing number of intervention studies have built up the evidence of the cardiovascular and metabolic benefits of the intake of these compounds in humans. However, the results remain limited and contradictory, leading to the current controversy regarding the consistency and magnitude of the protective effects that these compounds may exert in humans [17,18,19,20]. A potential reasoning underlying this might be the interindividual variability in the response to dietary compounds and the possibility that these compounds may be specifically beneficial for some human subpopulations [21,22].

The main objective of the present systematic review was to critically evaluate the current level of evidence of the benefits of dietary supplementation with various plant-derived products containing mixed phenolic and polyphenolic compounds ((poly)phenols from here onwards) in the specific subpopulation of postmenopausal women with an increased risk for cardiometabolic diseases. We have specifically evaluated the effects of the (poly)phenol-containing products on the levels of some of the main biomarkers associated with MetS and CVDs risk: (1) glucose metabolism indicators (glucose, insulin, Homeostatic Model Assessment of IR (HOMA-IR)); (2) circulating lipids (T-C, LDL-C, HDL-C, TGs); (3) blood pressure (systolic blood pressure (SBP), diastolic blood pressure (DBP)); (4) several biomarkers of inflammation (CRP, TNF-α, IL-6, adiponectin, soluble vascular cell adhesion molecule 1 (sVCAM-1), soluble intercellular adhesion molecule 1 (sICAM-1), sP-Selectin, and sE-Selectin); and (5) several biomarkers of oxidative stress (oxidized LDL (Ox-LDL), isoprostanes, 8-hydroxy-deoxyguanosine (8-OHdG), thiobarbituric acid reactive substances (TBARS), superoxide dismutase (SOD), glutathione reductase (GSR), glutathione peroxidase (GPX), and lipid hydroperoxide (LPO)).

## 2. Materials and Methods

### 2.1. Research Question, PICO and Study Protocol

The research question was “what is the effect of the dietary supplementation with a source of (poly)phenol-rich products on cardiometabolic risk factors in postmenopausal women?” Different studies were reviewed according to the population, intervention, comparison, and outcome (PICO) criteria (Table 1).

The study protocol was executed as per the recommendations of “Preferred Reporting Items for Systematic reviews and Meta-analysis (PRISMA)” [23]. The table with the results of PRISMA items analysis has included in supplementary material (Table S1). The study was also registered in the “International Prospective Register of Systematic Reviews” (PROSPERO) (CRD42021285214).

### 2.2. Search Strategy

We carried out a comprehensive search of the literature from the last 20 years using the PubMed, Web of Science (WOS), and Google Scholar databases (search was updated up to October 2021). All of the references of the selected studies were additionally revised and those relevant for this review were also included. The search strategy was designed as a combination of the following search terms: #1 AND #2 AND #3 AND #4 AND #5 (#1 climacteric OR menopause OR menopausal OR postmenopausal OR woman OR female; #2 “phenolic compounds” OR (poly)phenols; #3 “clinical trials” OR “dietary intervention study”; #4 “cardiometabolic risk” OR “cardiovascular health” OR “metabolic syndrome” OR “blood pressure”; #5 cholesterol OR “lipid profile” OR “total cholesterol” (T-C) OR “high-density lipoprotein (HDL-C)” OR “low-density lipoprotein (LDL-C)” OR triglycerides (TGs) OR “ body mass index (BMI)” OR “body weight” OR “body weight gain” OR “body composition” * OR “inflammatory biomarkers” OR “oxidized LDL” (ox-LDL) OR isoprostanes OR 8-hydroxy-deoxyguanosine (8-OHGdG) OR “soluble vascular cell adhesion protein 1 (sVCAM-1)” OR “soluble intercellular adhesion molecule 1 (sICAM-1)” OR “tumor necrosis factor (TNF-α)” OR adiponectin OR “antioxidant activity” OR “oxidative stress” OR “thiobarbituric acid reactive substances (TBARS)” OR “lipid hydroperoxide (LPO)” OR “antioxidant enzymes” OR “superoxide dismutase (SOD)” OR “glutathione S-reductase (GSR)” OR “glutathione peroxidase (GPx)” OR “glutathione reductase (GR)” OR insulin OR “insulin sensitivity” OR “insulin resistance” OR “fasting glucose” OR “Homeostatic Model Assessment of insulin (HOMA)” OR “glycated hemoglobin” OR HbA1c OR OR “systolic pressure” OR “diastolic pressure”).

### 2.3. Inclusion and Exclusion Criteria

We included only studies in English that met the following criteria: (1) randomized clinical trials (RCTs) or intervention studies with a placebo or control group (or any other comparative group such as high *vs* low doses); (2) study population constituted by postmenopausal women with amenorrhea for at least 12 months and who did not follow HRT; (3) the intervention arm was designed to test the effect of (poly)phenols as part of the diet either in the form of foods, extracts or supplements; and (4) they evaluated the effects on some of the following cardiometabolic biomarkers: glucose metabolism (glucose, insulin and HOMA-IR, lipid profile (T-C, HDL-C, LDL-C and TGs), SBP and DBP, inflammatory biomarkers (TNF-α, IL-6, CRP and adiponectin), endothelial cell adhesion molecules (sVCAM-1, sICAM-1, and selectin) and oxidative stress biomarkers (ox-LDL, isoprostanes, 8-OHdG, TBARS, LPO, SOD, GSR, GPx). Studies that did not meet these inclusion criteria were eliminated.

### 2.4. Data Extraction and Management

Data extraction was performed using a specifically designed Excel form. Extraction was carried out in duplicate by four authors, independently, and cross-checked by a fifth author. Extracted data included: (i) publication details (tittle, year of publication, authorship, journal name city/country); (ii) participants’ characteristics (age, ethnicity, health status, menopausal status, BMI, use of medication); (iii) study setting (total number of participants included in the study and in the analysis, design (cross-over or parallel), washout duration, treatment duration, number of arms and description, number of participants located in each arm and completing the study, composition and characteristics of the treatment and of the placebo/control, dosage); and (iv) information on available outcomes (type of sample, results before and after intervention for the control and treatment groups as well as changes in the variables estimated as the difference between the treatment group and the control group (“effect size”), *p*-value). Lipid and glucose levels were all converted to mg/dL. Results were generally presented as mean values and standard deviation (SD) unless otherwise stated. Where the standard error (SE) was reported, this was converted to SD. The “effect size”, the consistency of the direction of the changes, and the consistency of the statistical significance were compared across the studies. We additionally estimated data variability for the results of each group by calculating the coefficient of variation (CV%).

### 2.5. Assessment of the Risk of Bias

A systematic assessment of the risk of bias for each of the included studies was based on the Cochrane Collaboration measurement with some modifications [24]. The specific items used for the assessments of each study are those used in a previous meta-analysis [19]: (1) selection bias—random sequence generation, allocation concealment (in each item, yes = 1; no = 0, unclear = 0); (2) performance bias—blinding (yes = 1 for each participants, researchers and statisticians, no = 0, unclear = 0), measurement of compliance (1 for biomarker measure, 0.5 if compliance information was collected by counting non used capsules or recipients, or by self-reporting, 0 if no measurement of compliance was carried out or the information is insufficient); (3) attrition bias–flow of participants (1 if flow of participants is explained in detail, including number of withdrawals and reasons, 0 if there is no information or insufficient information); and (4) other bias—baseline comparability between test and control groups (yes = 1, no = 0, unclear = 0), data report (1 if pre and post data or change is reported in table with central measure and spread for placebo and treatment groups, and number per group, 0 if anything is missing), industry funding (0 if any commercial source provided some or all monetary funding for the trial, if a company carried out a study “in house”, if any of the authors was employed by a relevant industry or if it was unclear that there was any kind of industry funding, 1 if there was no funding from industry or if the only involvement of a company was to provide any ingredient for the intervention). Studies were rated as low risk of bias when total score was ≥8 and ≤10, moderate risk of bias when total score was ≥5 and ≤8 and high risk of bias when total score was <5 (Table S2).

## 3. Results

### 3.1. Study Selection

**Figure 1:**
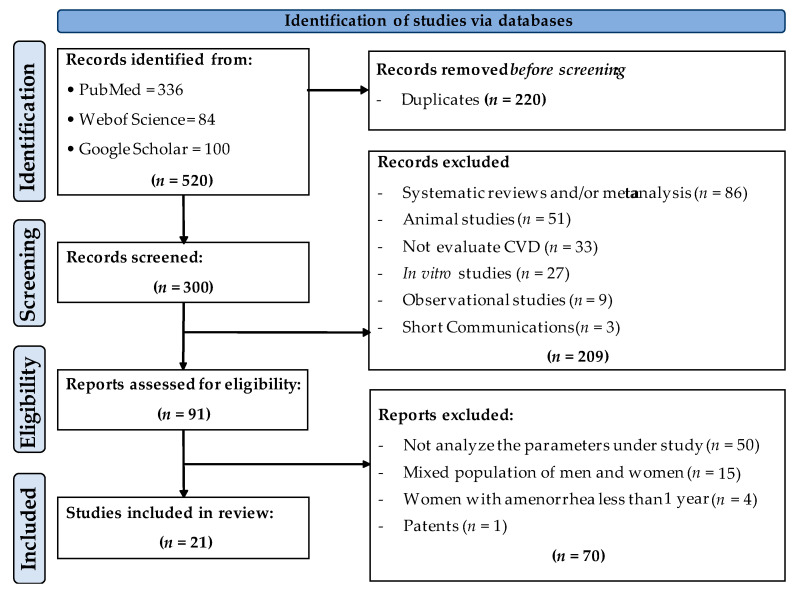
Flowchart of the study selection process.

The study selection process is shown in Figure 1. In the first identification phase, a total of 520 articles were obtained (336 from PubMed, 84 from Web of Science and 100 from Google Scholar), of which 220 were eliminated (duplicates). In the second screening phase, of the 300 articles identified as original, 209 were eliminated since they were systematic reviews and/or meta-analyses, animal studies, studies that did not evaluate cardiovascular disease parameters, in vitro cell culture studies, observational studies and/or short communications. Thus, a total of 91 articles were further evaluated to determine their eligibility. In this phase, 70 studies were eliminated since they did not analyze the main variables under investigation, and/or they were carried out in a mixed population (men and women), or in women without amenorrhea for more than 12 months. There was also a patent excluded at this stage. A total of 21 articles were included in the present review.

### 3.2. Description of the Characteristics of the Studies Finally Included in This Review

Table 2 presents a summary of the main characteristics of the studies included in this systematic review. All of the selected studies evaluated the impact of mixed (poly)phenols intake on cardiovascular risk factors. The studies were conducted following a parallel or crossover design, with a placebo or control group and a treatment group constituted by a (poly)phenol-rich product either in the form of food or extract.

Most studies were conducted in ‘healthy’ (no specific reported pathologies or conditions) postmenopausal 40–62 years old women and generally with an elevated BMI (i.e., obese and/or overweight). Only in a few studies were the participants diagnosed with dyslipidemia [25,26,27], osteopenia [28], hypertension [29,30], or MetS [31]. No specific pharmacological treatment was indicated except for the MetS sample population [31], in which the participants were under treatment with antihypertensive agents.

The number of participants included in each group (placebo/control or treatment) was found to be around 37; the smallest group was formed by 7 individuals [32], while the largest group was constituted by 120 participants [33]. With respect to the duration of the intervention, on average, most of the studies were carried out for six months, with the minimum intervention period being two weeks [34] and some studies lasting for up to a year [28,33,35]. Regarding the intervention arm with the (poly)phenol-containing products, we found a high heterogeneity, both for the format (foods, beverages, extracts, or capsules) as well as for the (poly)phenolic composition. Some studies investigated the effects of foods such as chocolate, plums, apples, blueberries, and grapes, and beverages such as red wine, beer, and cocoa. These foods were all rich mainly in flavanols (flavan-3-ols and procyanidins), anthocyanins, flavonols, and/or hydroxycinnamic acids derivatives. On the other hand, several other studies tested different capsules containing extracts of isoflavones and/or flavanols (flavan-3-ols and procyanidins). In addition, the daily dose of (poly)phenols ranged from 43 mg/day [25] to 1315 mg/day [33] with a mean value of 560 mg/day. According to the risk of bias, most of the studies (~81%) were classified as studies with a moderate risk of bias (score ≥5.0 and ≤8.0), while the rest of them (~19%) were considered as having a high risk of bias (score < 5).

**Table 2:** Main characteristics of the studies included in this review evaluating the impact of the consumption of (poly)phenol-rich products on cardiometabolic risk factors in postmenopausal women with >1 year since the last menstrual period.

References	Study Design	Reported Health Status/ BMI (kg/m^2^)	Mean Age (Years)	Treatment/Supplementation/ Dose (poly)Phenols/Duration	Risk of Bias (Score) *
Cheng et al., 2004 [36]	Double-blind parallel P (*n* = *11*); T (*n* = *17*)	Healthy BMI P: 23 ± 2.0; T: 22 ± 2.6	P: 58 ± 6.0; T: 61 ± 8.9	P: Estrogen capsules 0.625 mg T: Isoflavone capsules (primrose oil, daidzein and genistein (*w*/*w*/*w* ratio 1:1:3) Dose (poly)phenols: 100 mg/day; Duration: 180 days	7.0
Sathyapalan et al., 2018 [37]	Double-blind parallel P (*n* = *60*); T (*n* = *60*)	Healthy BMI P: 27 ± 7.0; T: 27 ± 4.6	P: 52; T: 52	P: Soy protein free of isoflavones T: Soy protein with isoflavones Dose (poly)phenols: 66 mg/day; Duration: 180 days	7.0
Myasoedova et al., 2016 [35]	Double-blind parallel P (*n* = *71*); T (*n* = *56*)	Healthy (mixed population of asymptomatic women with hypertension, and high cholesterol were included) BMI P: 27 ± 3.8; T: 27 ± 4.0	P: 65 ± 6.0; T: 65 ± 7.0	P: Placebo capsules T: Mixed herbal preparation rich in isoflavonoids, containing tannins from grape seeds, green tea leaves, hop cone powder and garlic powder Dose(poly)phenols: 283 mg/day; Duration: 365 days	6.0
Wu et al., 2012 [38]	Double blind parallel, with three arms P (*n* = *32*); T1 (*n* = *37*); T2 (*n* = *34*)	Healthy BMI P: 29 ± NI; T1: 30 ± NI; T2: 29 ± NI	P: 58 ± 6.3; T1: 60 ± 6.4; T2: 62 ± 9.4	P: Placebo capsules T: EGCG and other catechins included EC, EGC, ECG, and GCG Doses (poly)phenols: T1: Low dose (400 mg/day); T2: High dose (800 mg/day); Duration: 160 days	5.0
Curtis et al., 2009 [39]	Double-blind parallel P *(n = 26);* T *(n = 26)*	Healthy BMI P: 24 ± 3.4; T: 25 ± 3.8	P: 58 ± 5.8; T: 58 ± 5.5	P: Placebo capsules T: Elderberry extract (anthocyanins) Dose (poly)phenols: 500 mg/day; Duration: 84 days	5.5
Zern et al., 2005 [40]	Single blind crossover P (*n* = *20*); T (*n* = *20*)	Healthy BMI P/T: 31 ± 4.6	P: 40 ± 8.5; T: 59 ± 7.5	P: Placebo capsules T: Lyophilized grape powder (flavanols, anthocyanins, quercetin, myricetin, kaempferol, and resveratrol) Dose (poly)phenols: 210 mg/day; Duration: 84 days	4.0
Chai et al., 2012 [41]	Single blinded to researcher, parallel T1 (*n* = *55*); T2 (*n* = *45*)	Healthy BMI T1: 25 ± 4.1; T2: 25 ± 4.6	T1: 56 ± 5.0; T2: 58 ± 4.0	T1: Dried apples (75 g) T2: Dried plums (100 g) Dose (poly)phenols: NI; Duration: 180 days	6.5
Al-Dashti et al., 2019 [34]	Open-label crossover T1 (*n* = *27*); T2 (*n* = *27*)	Healthy BMI T1: 25 ± 6.1; T2: 24 ± 6.8	T1: 59 ± 5.2; T2: 59 ± 5.2	Prunes (*Prunus domestica* L) T1: Low dose (14 g of dried prunes) T2: High dose (42 g of dried prunes) Dose (poly)phenols: NI; Duration: 14 days	4.5
García-Yu et al., 2020 [42]	Single blinded to researchers, parallel C (*n* = *66*); T (*n* = *71*)	Healthy BMI C: 27 ± 3.1; T: 26 ± 3.8	C: 58 ± 3.8; T: 57 ± 3.5	C: Not receiving any interventionT: Dark chocolate, 99% cocoa (10 g). (Poly)phenols: protocatechuic acid, catechins, procyanidins, quercetin Dose(poly)phenols: 65.4 mg/day; Duration: 180 days	6.5
García-Yu et al., 2021 [43]	Single blinded to researchers, parallel C (*n* = 61); T (*n* = 67)	Healthy BMI C: 25 ± 3.1; T: 26 ± 3.8	C: 57 ± 3.8; T: 57 ± 3.6	C: Not receiving any intervention T: Dark chocolate, 99% cocoa (10 g). (Poly)phenols: protocatechuic acid, catechins, procyanidins, quercetin Dose (poly)phenols: 65.4 mg/day; Duration: 180 days	6.5
Estévez-Santiago et al., 2019 [44]	Open label, parallel, with three arms T1 (*n = 26)*; T2 (*n = 23)*; T3 *(n = 23)*	Healthy BMI T1: 25 ± 2.8; T2: 25 ± 3.3; T3: 25 ± 2.7	T1: 60 ± 6; T2: 58 ± 6; T3: 60 ± 5	T1: Xanthophylls (6 mg lutein + 2 mg zeaxanthin/day) T2: Anthocyanins (60 mg/day) T3: Anthocyanins (60 mg/day) and xanthophylls (6 mg lutein + 2 mg zeaxanthin/day); Doses (poly)phenols: 60 mg/day; Duration: 240 days	4.0
Trius-Soler et al., 2021 [32]	Open label, controlled parallel, with three arms C (*n* = *14*); T1 (*n* = *16*); T2 (*n* = *7*)	Healthy BMI C: 27 ± 4.4; T1: 25 ± 3.7; T2: 30 ± 9.0	C, T1, T2: 45–70	C: Not receiving any intervention T1: Beer with alcohol (330 mL/day with 14 g of etanol) T2: Beer without alcohol (660 mL/day) Doses (poly)phenols: T1: 0.359 mg/day of prenylflavonoids; T2: 0.259 mg/day of prenylflavonoids; Duration: 180 days	4.5
Filip et al., 2015 [28]	Double blind parallel P (*n* = *21*); T (*n* = *27*)	Osteopenia (mix cholesterol levels) BMI P: 28 ± 4.0; T: 26 ± 4.3	P: 59 ± 5.6; T: 60 ± 4.4	P: Placebo capsules T: Calcium supplement (1000 mg Ca) and olive extract (250 mg/day) Dose: >100 mg oleuropein; Duration: 365 days	7.5
Wang-Polagruto et al., 2006 [25]	Double-blind parallel T1 (*n* = *16*); T2 (*n* = *16*)	Dyslipidaemia (high cholesterol) BMI T1: 25 ± 3.2; T2: 25 ± 4.0	T1: 55 ± 6.8; T2: 58 ± 8.8	Flavanol cocoa beverage T1: Low flavanol dose T2: High flavanol dose Doses (poly)phenols: T1: 43 mg/day; T2: 446 mg/day; Duration: 42 days	5.5
Naissides et al., 2006ab [26,27]	Open label, parallel, with three arms C (*n* = *16*); T_1_ (*n* = *15*); T_2_ (*n* = *14*)	Dyslipidaemia (high cholesterol) BMI C: 27 ± 4.8; T_1_: 26 ± 6.0; T_2_: 26 ± 3.5	C: 59 ± 5.6; T_1_: 58 ± 4.9; T_2_: 58 ± 5.0	C: Water (400 mL) T_1_: Non-alcoholic red wine (400 mL) T_2_: Alcoholic red wine (400 mL) Doses (poly)phenols: 1000 mg/day red wine (poly) phenols, each treatment group; Duration: 42 days	6.0 /5.0
Aubertin-Leheudre et al., 2008 [45]	Double-blind parallel P (*n* = *18*); T (*n* = *21*)	Obese BMI P: 31 ± 4.5; T: 33 ± 4.8	P: 57 ± 5.6; T: 58 ± 5.2	P: Placebo capsules T: Isoflavone capsules containing 70 mg isoflavones extracted from natural soy (44 mg daidzein, 16 mg glycitein, and 10 mg genistein) Dose (poly)phenols: 70 mg/day; Duration: 180 days	5.5
Dostal et al., 2016 [33]	Double-blind parallel P *(n = 120)*; T *(n = 117)*	Obese and overweight BMI P: 28 ± 2.7; T: 29 ± 3.0	P: 61 ± 5.2; T: 61 ± 4.9	P: Placebo capsules T: Decaffeinated green tea extract Dose (poly)phenols: 1315 mg catechins/day; Duration: 365 days	7.5
Johnson et al., 2015 [29]	Double-blind, parallel with two arms P (*n* = *20*); T (*n* = *20*)	Seated blood pressure ≥125/85 mm Hg but ≤160/90 mm Hg BMI P: 33 ± 6.5; T: 30 ± 5.9	P: 57 ± 4.8; T: 60 ± 4.6	P: Placebo powder T: Blueberry powder (22 g) Dose (poly)phenols: Phenolics (845 mg/day) and anthocyanins (469 mg/days); Duration: 56 days	7.0
Johnson et al., 2017 [30]	Double-blind parallel with two arms P (*n* = *20*); T (*n* = *20*)	Pre- and stage 1-hypertension BMI NI	P, T: 45–65	P: Placebo capsules T: Blueberry powder (22 g) Dose (poly)phenols: Phenolics (845 mg/day) and anthocyanins (469 mg/days); Duration: 56 days	7.5
D’Anna et al., 2014 [31]	Open parallel P (*n* = *21*); T (*n* = *22*)	MetS BMI P: 34 ± 3.9; T: 32 ± 3.8	P: 56 ± 4.8; T: 56 ± 3.8	P: Placebo powder T: Cocoa (poly)phenols (30 mg), soy isoflavones (80 mg) and myoinositol Dose (poly)phenols: 110 mg/day; Duration: 180 days	5.5

P, placebo; C, control; T, treatment; T1, treatment low dose; T2, treatment high dose; BMI, body mass index; EGCG, epigallocatechin gallate; EC, epicatechin; EGC, epigallocatechin; ECG, epicatechingallate; GCG, gallocatechin gallate; NI, not indicated; MetS, metabolic syndrome. * Risk of bias score: low risk (≥8 and ≤10), moderate (≥5 and <8), high risk (<5).

### 3.3. Analysis of the Results of the Selected Studies

All the studies included in this review were thoroughly examined to register for the changes in the biomarkers associated with cardiometabolic risk. Data were compiled and included in the supplementary material as follows: fasting glucose, insulin and HOMA-IR (Table S3), lipid plasmatic profile (T-C, LDL-C, HDL-C and TGs) (Tables S4–S7), blood pressure (both SBP and DBP) (Table S8), inflammation and endothelial cell adhesion molecules (CRP, TNF-α, IL-6, adiponectin, sVCAM-1, sICAM-1, sP-Selectin, sE-Selectin) (Table S9), oxidative stress biomarkers (Ox-LDL, isoprostanes, 8-OHdG, TBARS and LPO) and antioxidant enzymes (SOD, GSR and GPX) (Table S10). These tables, include general information about the type and design of the study, as well as the specific results attained for each biomarker in: (i) the placebo, control or any other comparator group before and after intervention; (ii) the treatment group with the (poly)phenol-rich product before and after intervention, (iii) the comparison between control and treatment groups. To add in the reading and comprehension of the results, we have included within the manuscript summary Table 3, Table 4, Table 5 and Table 6 that present an overview of the study and the results with a focus on several critical issues: (i) the variability of the results estimated as the CV(%) for each group; (ii) the size and direction (+ or −) of the effects as the range of differences between placebo/control and treatment groups; (iii) the consistency of the change to indicate whether all the studies show the same tendency or not; and (iv) the consistency of the reported statistical significance of the change (i.e., whether all results were significant or not).

### 3.4. Changes in the Glucose Homeostasis Indicators

Table S3 gathers the results from the studies that evaluated the impact of (poly)phenol-rich products on glucose-related markers in postmenopausal women. Among those, fasting glucose, fasting insulin, and HOMA-IR index were the most repeatedly investigated whereas none of the studies reported values of glycated hemoglobin. The overall results are summarized in Table 3. As indicated by the values of the CV%, there was, in general, a high variability in the results with values ranging from 6% to 39% for fasting glucose, 11% up to >100% for insulin, and 27% up to >100% for the HOMA-IR index. We found a lack of consistency in the reported effects with both increases or decreases of glucose and insulin following the intake of the different (poly)phenols-rich products. Of the 13 trials included in this section, only two of them reported a significant reduction in fasting glucose and insulin following the consumption of a green tea extract, rich mostly in flavan-3-ols [38], and the consumption of snack bars of soy protein with isoflavones [37] compared to a placebo group. In the latter study, the HOMA-IR index was also significantly reduced after the consumption of soy protein with isoflavones. A third study performed with mix cocoa (poly)phenols and soy isoflavones [31], significantly reduced glucose in the treatment group compared to placebo group. In the rest of the trials, no significant differences were found between groups in any of the glucose-related markers.

### 3.5. Changes in the Lipid Profile

Tables S4–S7 show the changes for the lipid profile (T-C, LDL-C, HDL-C, and TG) in postmenopausal women after intervention with the different products rich in (poly)phenols. The results are summarized in Table 4. Overall, a great variability of the results was also observed, as indicated by a CV (%) that oscillates between 10% and 48% for T-C, 13% and 92% for LDL-C, 10% and 66% for HDL-C and 10% and 80% for TGs. Regarding the effect size, both positive and negative changes were observed in all these variables after supplementation with some studies reporting significant changes, whereas in other studies the results were not significant. Thus, there was no consistency either in the reported changes or the statistical significance.

### 3.6. Changes in Blood Pressure (BP)

Regarding the changes in blood pressure, dissimilar results were also observed among the different intervention studies (Table S8). Data also show a high variability with CV (%) values ranging from 7% up to 29%. The reported changes were weak, and generally not statistically significant (Table 5). Overall, the intake of (poly)phenols-rich products has no clear effect in the BP of postmenopausal women. Only 2 out of 12 studies achieved statistically significant (*p* < 0.05) group × time interactions. Johnson et al. [29] reported a significant decrease of SBP (−8.0 mmHg) and DBP (−7.0 mmHg) after the intake of blueberry powder for 56 days, compared to placebo. In addition, Sathyapalan et al. [37] also observed a significant reduction of SBP (−2.5 mmHg) in subjects consuming soy isoflavones for 180 days.

### 3.7. Changes in the Inflammatory, Endothelial Function and Oxidative Stress Biomarkers

The results of the changes in the inflammatory (CRP, TNF-α, IL-6, and adiponectin) and endothelial cell adhesion (sVCAM-1, sICAM-1, sP-Selectin, sE-Selectin) biomarkers (Table S9) as well as those of the oxidative stress biomarkers (Ox-LDL, isoprostanes, 8-OHdG, TBARs and LPO) and antioxidant enzymes (SOD, GSR and GPX) (Table S10) following intervention with various (poly)phenol-containing products in postmenopausal women are summarized in Table 6.

There was also, in general, a high variability of the data for the inflammatory molecules with CV (%) values ranging from ~15% up to >100%. Regarding the effect size, we also found a lack of consistency with studies reporting either a small increase or decrease of the levels of the selected inflammatory biomarkers. Further, the statistical significance of those results is very limited and inconsistent. Overall, there is not a noticeable, consistent and significant alteration of the levels of CRP, TNF-α, IL-6 or adiponectin following intervention with mixed (poly)phenols in postmenopausal women. Regarding the endothelial adhesion markers, we only found one study reporting changes in the levels of the soluble molecules sVCAM-1, sICAM-1, sP-selectin, and sE-selectin in postmenopausal women following intervention with cocoa (poly)phenols. The results include only a significant effect size in the levels of sVCAM-1 with highly variable results [25].

Among the total of selected studies, only four of them reported the analysis of some biomarkers of oxidative stress [29,30,40,41]. As already indicated for other biomarkers, we observed a high variability in the data with CV (%) values ranging from 5% up to >100% (Table 6). There were no significant differences between Ox-LDL, isoprostanes, 8-OHdG, TBARS and LPO concentration in control/placebo/comparator and treatment groups. However, TBARS increased around 38% and 12%, after the intake of 845 mg of phenolics, during 28 and 56 days, respectively [30]. Chai et al. [41] reported a significant decrease in serum LPO in both DA and DP experimental groups, before and after intervention during 365 days (37% and 34%, respectively) but with no significant differences between groups. It should be noted that in this study, the effect was compared between the two different products. On the other hand, the activity of antioxidant enzymes increased both in the control and treatment groups after the intervention period and no significant effect was observed when treatment group was compared with the control or placebo groups (Table S10). The oxidative stress biomarkers were only reported in one study and thus, the consistency of the change and the consistency of the statistical significance were not applicable.

## 4. Discussion

During menopause, a fall in the levels of estrogens contributes to the development of metabolic alterations such as the increase of BMI and adiposity, and/or disorders of the lipid and carbohydrate metabolism that can predispose to the development of CVDs and T2DM, and affect the length and quality of life in women [46]. The current HRT improves menopausal symptoms and modifies cardiovascular risk factors, but the results are contradictory [47], and the benefits do not exceed the adverse effects associated to the administration of estrogens and progestin [10]. Thus, HRT is not recommended as an effective therapeutic strategy against cardiometabolic disorders. Instead, lifestyle changes towards moderate exercise and a healthier diet continue to be a first-choice recommendation to combat these disorders in postmenopausal women [46]. Among the dietary recommendations, the Mediterranean diet (MD) and many of their foods and components are considered of great benefit against menopausal metabolic alterations. Of specific interest are some of the MD constituents such as vitamins, polyunsaturated fatty acids, carotenoids and (poly)phenolic compounds. (Poly)phenols are abundant in plant-based foods and specific types of compounds such as flavonoids (flavonols, isoflavones, flavanols) and phenolic acids (chlorogenic acid) which are found, in significant amount, in a diversity of foods and beverages (berries, olives, grapes, soybean, tea, cocoa, wine) have been recommended to counteract the postmenopausal changes [48]. Nevertheless, a critical revision of the benefits against the cardiometabolic disorders in postmenopausal women of (poly)phenols containing foods and products was lacking.

(Poly)phenols have been investigated and reported as bioactive compounds with multiple beneficial effects including antioxidant and anti-inflammatory activity as well as modulatory properties of cardiometabolic risk associated biomarkers (i.e., body weight, lipids and glucose levels, SBP and DBP). All these effects were first revealed in pre-clinical models with different mechanisms of action proposed at different body sites (host organs, gastrointestinal tract, microbiota) and the responsible molecules may be the original plant compounds, and/or their derived metabolites [49]. These compounds may affect the digestion and absorption of lipids and carbohydrates, alter the microbiota composition and its metabolic capacity [49] and/or interfere with specific host tissue metabolic pathways [6]. In humans, an increasing number of studies support the cardiometabolic modulatory effects of dietary (poly)phenols and their potential to battle against the risk factors associated with CVDs [13,50,51,52]. In the past few years, several meta-analyses have collected the evidence supporting the capacity of a diversity of (poly)phenols to moderate the cardiometabolic risk factors in the general adult population. Flavonols (abundant in vegetables and some berries) have been reported to significantly reduce T-C, LDL-C, TGs, glucose and BP (both SBP and DBP) as well as to increase HDL-C [53]. Flavanol-containing tea, cocoa and apple show similar effects and significantly reduce BMI and waist circumference [17]. Equally, anthocyanin- and ellagitannin-containing products (berries, nuts) reduce T-C with nuts and berries yielding more significant effects than pomegranate and grapes. BP was also reduced by the two main sources of anthocyanins, berries and red grapes/wine, whereas waist circumference, LDL-C, TGs, and glucose were most significantly lowered by the ellagitannin-products, particularly by nuts. Additionally, HDL-C was significantly increased with the intake of nuts [19]. A more recent meta-analysis also shows that the consumption of (poly)phenols in general contributed to reduce fasting glucose levels and glycated hemoglobin (Hb1Ac) whereas the levels of insulin and HOMA-IR were not significantly altered [20].

We herein present the results of the evaluation of the cardiometabolic effects of some of these (poly)phenol-containing foods and products in the specific group of postmenopausal women with at least one year of menopause symptoms and not taking HRT. We analyzed the effects on glucose, insulin, HOMA-IR, lipid levels, SBP and DBP, as well as on some inflammatory, cell adhesion, and stress oxidative biomarkers. All of the examined studies were conducted for sufficiently ample periods of time (≈6 months on average, ranging from 14 days [34] up to 1 y [28,33,35] and with sufficiently high doses (from 43 mg/d [25] to 1315 mg/d [33] of mix (poly)phenols, mostly flavan-3-ols, procyanidins, anthocyanins, isoflavones and hydroxycinnamic acids present in chocolate, plums, apples, grapes and wine. In contrast to the benefits reported for the general population [17,19,20,53], the results of our analysis suggest a lack of evidence for beneficial cardiometabolic regulatory effects of the (poly)phenols in postmenopausal women. We have found that: (i) the different biomarkers analyzed displayed a large variability (as indicated by the high values of the CV (%), often >100%; (ii) the magnitude of the changes (‘effect size’) was generally small (i.e., from a few units to even less than one unit); (iii) the statistical significance of the differences between treated and control groups was also limited and inconsistent; and (iv) the changes in these biomarkers were not consistent (i.e., both increases or decreases were reported after the intervention with the different (poly)phenol-containing foods and products). Nonetheless, important differences between the studies, and the heterogeneity of some of the characteristics of the participants might have largely contributed to the lack of consistent and significant results.

From a methodological point of view, different elements may have affected the results and precluded a clear detection of potential benefits: (i) the small number of studies that could be selected and included in this review (21); (ii) the limited size of the sample populations involved in the studies, mostly between 15 and 20 per group (with a minimum size of 7 [32] to a maximum size of 120 participants per group [33]; (iii) differences in the source, nature and matrix of (poly)phenols (extracts, foods, beverages); (iv) presence of other constituents (i.e., fiber) which may have interfered with the effects of the (poly)phenolic compounds [54,55]; (v) not well designed control or placebo groups that should differentiate only in the (poly)phenols content [25,34,41,44]; and (vi) other general methodological specific issues such as the type of biological sample used (plasma, serum, fasting) or the analytical procedure applied. General recommendations regarding the RCTs have been thoroughly described indicating the need to improve the overall quality of the study design [56] and the presentation of the results in humans following intervention with bioactive compounds [57].

Along with the sex and age of the participants, a number of other relevant factors such as ethnicity, the gut microbiota composition and function, (epi)genetic make-up and/or the metabolic and absorption capacity (bioavailability) of the (poly)phenols have been indicated for their potential impact on the human responses to dietary (poly)phenols [21,58]. The women included in all the studies reviewed here were between 40 (early menopause) and 62 (late menopause) years old which could indeed have an effect on the variability of the results. The bioavailability of the (poly)phenols also varies among the different classes of compounds and between individuals. These compounds are normally rapidly metabolized and excreted and thus low concentrations are generally detected in plasma, urine and tissues which may partially explain the limited effect size [59]. In addition, the cardiometabolic health status (obesity, dyslipidemia, etc.) of the individuals can also influence largely the response to (poly)phenols. As already stated, postmenopausal women usually experience an increase in BMI and abdominal adiposity as well as in LDL-C and TGs levels (10–15% higher) and BP with slightly lower HDL-C levels. The postmenopausal participants included in this review were mostly described as overweight and/or obese but otherwise healthy women. Our analyses confirmed that at baseline most of them exhibited: (i) normal levels of glucose (<100 mg/dL) [60] with only two studies [31,36] displaying mean values that indicated impaired glucose values (100–125 mg/dL); (ii) average normal levels of TGs (<150 mg/dL) [61] except for one study where a mild hypertriglyceridemia was manifest [31]; (iii) high levels of HDL-C (>60 mg/dL) with only four studies reporting moderate baseline values and none of them exhibiting low values [28,31,32,45]; and (iv) normal DBP and SBP (<85 mm Hg and <130 mm Hg, respectively) [62] except for five studies [25,29,31,35,39] where SBP reached values slightly higher (130–140 mm Hg). Regarding LDL-C, values ranged from near/above optimal (100 to 129 mg/dL) [61] to borderline high (130 to 159 mg/dL) with one study [28] reaching rather high values (167 to 174 mg/dL) displaying (although not clearly reported) signs of moderate hyperlipidemia in many of the participants. Nevertheless, as already stated, the baseline CV (%) values were very high supporting the evidence of a large initial interindividual differences in the levels of these biomarkers for the participants, all of which might have also contributed to preclude the detection of effective benefits of these compounds in the postmenopausal women.

Our results show a general lack of significant and consistent effects of the intake of mix (poly)phenols with both increases and decreases reported for all the different biomarkers tested, and an ample variation in the size of these effects. In particular, the effect size in LDL-C ranged from an increase of +18.1 mg/dL following 90 d of consuming encapsulated isoflavones (100 mg/day) [36] to a significant reduction of −16.1 mg/dL (*p* = 0.016) after the intake of dealcoholized beer rich in phenylpropanoids (0.259 mg/day) during 180 d [32] or the intake of capsules containing green tea extract rich in flavanols (400 mg/day and 800 mg/day) for 60 d which showed a significant reduction in LDL-C (*p* = 0.021) for the two doses (7.4 and 8.1 mg/dL, respectively) [38]. The size of these effects would only achieve the targets associated with a reduction of CVDs risk (i.e., <70 mg/dL for patients at very high risk, or <115 mg/dL for low-risk individuals [63] should the LDL-C baseline values of the participants were in the near/above optimal ranges [61] but not for very high levels of LDL-C. It has also been estimated that small reductions (1 to 5 mm Hg) in SBP can have a substantial impact on disease prevention and reduce the relative risk of CVDs [64,65]. The recent American College of Cardiology (ACC)/American Heart Association (AHA) hypertension guideline and the European Society of Hypertension (ESH)/European Society of Cardiology (ESC) guideline recommended the lowering of target SBP/DBP to less than 130/80 mm Hg in most hypertensive subjects [61,66,67,68]. In postmenopausal women, SBP was not consistently reduced by the intake of (poly)phenols and both increases and decreases were reported. Nevertheless, some of the studies that indicated a decrease in SBP (even if not significant) reported reduction values between 2.0 and 11.0 mm Hg [26,29,32,37,44,45], suggesting that if these reductions were confirmed, the long term intake of (poly)phenols could have some impact in the cardiometabolic health of postmenopausal women but that this reduction would be more effective in women with only initial pre-hypertension [68] such as the significant reduction in SBP following the intake of blueberry powder containing mix (poly)phenols reported by Johnson et al. [29]. Overall, it is tempting to hypothesize that (poly)phenols might be of help principally at early stages of the cardiometabolic disorders appearing in postmenopausal women but further studies are needed to confirm this.

Regarding other biomarkers such as insulin and HOMA-IR, inflammatory molecules (CRP, TNF-α, IL-6, adiponectin), endothelial cell adhesion molecules (sICAM-1, sVCAM-1, sP-Selectin, sE-Selectin), and oxidative stress (oxLDL, antioxidant enzymes, isoprostanes, etc) biomarkers, there appears to be broad ranges of what has been reported as normal or healthy levels, and disease cut-off criteria have not been yet clearly established for all. For example, healthy values of CRP have been indicated to range from 0.6 to 10 mg/L [69,70,71,72,73,74,75,76], but a 3.0 mg/L level has been reported as the criteria for increased risk of MetS [77]. Our analysis show that the changes in CRP in response to the (poly)phenol-containing products in the postmenopausal women were small and varied from −0.46 to +1.0 mg/L. A reduction of −0.46 mg/L constitutes a ~15% change in comparison with the 3 mg/L cut-off level but only a ~5% change as compared with the average top normal value of 10.0 mg/L. Similarly, the normal circulating levels of TNF-α, another important inflammatory biomarker, exhibit a very high variability which expand from non-detectable up to 76 pg/mL, though, most values seem to be placed between 0.0 and 16 pg/mL [72,73,74,78,79,80,81,82,83,84,85,86,87,88]. A potential reduction in TNF-α of −3.0 pg/mL in response to the (poly)phenols intake constitute a ~20% change in comparison with a top normal level of 16 pg/mL but only a ~4% change in comparison with a maximum normal value of 76 pg/mL. In individuals with MetS, TNF-α levels can rise up to 140 pg/mL [89] and thus, the small potential changes attributed to (poly)phenols may contribute very little to reducing the levels of this inflammatory biomarker. These data suggest that when reporting the responses of biomarkers to (poly)phenols consumption, it is essential to try to understand and describe the relevance of the change within the context of the interindividual variability and the differences in the values between health and disease for each specific biomarker.

Of note, and in relationship with the quality of the data reporting of the RCTs, it is worth mentioning here the issue of the ‘units’ used to present the results of the different biomarkers. CRP is commonly expressed in mg/L, IL-6 and TNF-α in pg/mL, adiponectin in µg/mL and the adhesion molecules in ng/mL, however, reviewing the studies included here we noticed that, in some reports, different units had been used for some of the biomarkers (Table S8). When we converted those results to express them using the same most common units, some of the effects became either a thousand times higher such as for CRP [30] or lower for IL-6 [40] than the most habitual reported values, which may imply either a potential error or rather abnormal values for postmenopausal women. Along these lines, we have observed similar discrepancies in the insulin data [42,43]. These authors reported insulin values in mg/dL instead of µIU/mL (most commonly used) and, when those values were converted to µIU/mL, the results were not reliable (Table S2). It is thus of upmost importance for future studies investigating the levels and changes in these biomarkers that results are all indicated using the same most common units and that results are compared with well-established reference ranges to detect abnormal results and differentiate true effects.

Oxidative stress is closely associated with chronic inflammation and high contents of reactive oxygen species (ROS) are associated with obesity and MetS [90]. Considering the higher incidence of these disorders in postmenopausal women, oxidative stress biomarkers should also be investigated in dietary intervention studies carried out in this specific subpopulation. We only found four studies in which several oxidative stress biomarkers (ox-LDL, isoprostanes in serum and urine, 8-OHdG, TBARs, LPO and antioxidants enzymes) were analyzed following intervention with (poly)phenol-rich products (Table S9). As stated for other previously examined biomarkers, the results showed a lack of clear effects of the (poly)phenol-containing products on oxidative stress biomarkers, a high variability for the observed values and the difficulty in describing the significance of these results within the context of the physiological and pathological levels of these biomarkers. For example, ox-LDL, a well-known oxidative biomarker associated with the development of CVDs, has been indicated to be increased with BMI in both sexes as well as with other metabolic alterations, such as T2DM and MetS. However, distinct levels between healthy normal individuals and obesity or cardiometabolic disease have not yet been clearly defined [91]. In this fashion, the ox-LDL normal values for adult people with no coronary heart disease (CHD) has been indicated to be 8.0 ± 4.7 mg/L, whereas adult people with CHD exhibited higher values, 13.4 ± 8.1 mg/L [91]. Using a different analytical protocol, these same authors also reported the ox-LDL in arbitrary units (U/L) for the same groups, with mean values of 44 ± 13 and 54 ± 12 U/L, respectively, and propose a cut-off value of ox-LDL of >15 mg/L or 65 U/L. In a different study, the levels of ox-LDL in overweight men were reported to vary between 11 to 88 U/L [92]. In adult women (pre- and postmenopausal) without MetS the mean value of ox-LDL was found to be around 60 U/L, whereas in a similar group with MetS the values increased up to 67 U/L [93]. Ox-LDL values in premenopausal and postmenopausal women have been reported to be 40.9 ± 11.4 U/L and 56.7 ± 23.8 U/L, respectively [94], suggesting that postmenopausal women have a higher level of ox-LDL than premenopausal. All these data confirm the difficulty in establishing clear cut-off levels for this biomarker. We only found one study in which ox-LDL was measured following intervention with a mixture of (poly)phenols (phenolics and anthocyanins from blueberry) where a non-significant increment of 60 ng/mL was reported [30]. The conversion of this value to mg/L or U/L (assuming a conversion factor of 5 [91]) yields a change of only 0.06 mg/L or 0.3 U/L which indeed constitutes a rather small effect size in the context of the levels described for ox-LDL. For the other oxidative biomarkers investigated, we also found a considerable variability in the description of normal or reference values, i.e., isoprostanes (<50 pg/mL in serum and 1.4–1.6 ng/mg creatinine in urine) [93,95,96,97], TBARs (<1–60 nmoles/mL) [93,97,98,99], LPO (up to 500 nM) [100,101], SOD (3.5–12 U/mL) [102], GSR (10–70 U/mL) [103] or GPX (355–390 U/L [102], 40–50 U/g haemoglobin [104]).

Overall, and to further comprehend the differences in the responses to dietary (poly)phenols and the factors that influence these differences, one important issue to reflect on is the definition of such responses (i.e., what may be considered a ‘beneficial effect’ for each of the CVDs biomarkers investigated). Looking at the results of our analysis and of previous ones [19], it has become clear that the size of the effects of the dietary (poly)phenols is, in general, moderate to small and thus, it is important that the effect size is critically analyzed and the biological relevance established, so that future RCTs can reliably detect or disprove these small changes and their impact on cardiometabolic health. For this purpose, it is important to establish (i) the human interindividual variability in the levels of each of the biomarkers associated with CVDs risk and the multiple factors that influence this variability (i.e., type of population and country of origin, ethnicity, sex, age, health status); (ii) appropriate reference values associated with a normal or healthy status that may be differentiated from levels associated with cardiometabolic disease or altered-phenotypes (obesity, MetS, T2DM, hypercholesterolemia, hypertension); (iii) the factors that influence this difference; and (iv) how these changes can influence the long term reduction of CVD risk [105].

## 5. Conclusions

The RCTs carried out with different (poly)phenols rich products/foods and gathered in this review do not offer yet sufficient evidence for general or specific recommendations on the consumption of any of the tested mixed compounds to effectively moderate classic, inflammatory and/or oxidative stress biomarkers associated with a reduction of CVDs in the specific subpopulation of postmenopausal women. The lack of consistent and significant results may be partially due to the heterogeneity of the studies, including the diversity and complexity of the mixed compounds tested, as well as to the interindividual variability of the participants. Future studies should focus on improved study designs with appropriate placebos to clearly differentiate the effects of the (poly)phenolic compounds and larger samples of postmenopausal women with a well-characterized cardiometabolic status. Is also of upmost interest to correlate the potential effects with the metabolism and absorption of the compounds and to enhance our knowledge of the differences between health and disease for each specific cardiometabolic biomarker investigated as well as of the true beneficial effect size.

## Figures and Tables

**Table 1:** PICO of the study’s research question.

PICO Components	Determinants
Population	Postmenopausal women with amenorrhea for at least 12 months and who did not follow HRT
Intervention	Supplementation with (poly)phenol-rich products
Comparison	Placebo or control or any other comparative group (i.e., high vs. low doses of (poly)phenol-rich product)
Outcome	Cardiometabolic biomarkers: blood glucose/insulin, and HOMA-IR, blood lipids, SBP and DBP, and blood inflammatory biomarkers, endothelial cell adhesion molecules and oxidative stress biomarkers.

**Table 3:** Summary of the collected responses of the biomarkers-related to glucose metabolism to the daily consumption of mix (poly)phenols of different sources in postmenopausal women (for specific results in each study please refer to Table S3).

Markers of Glucose Metabolism	Study Characteristics				Effect (Change)			
	Type of Population	Source of (poly)phenols/(poly)phenols	Doses (mg/day)	Duration (days)	Variability of the Results (CV%)	Effect Size Range	Consistency of the Change ^1^	Consistency of the Statistical Significance ^2^
Glucose (mg/dL) (*n* = 12) [27,31,32,33,34,36,37,38,39,42,44,45]	Mix (healthy, obese, dyslipidaemia, MetS and overweight)	Snack bar (soy protein, mix isoflavones), chocolate 99% (procyanidins, epicatechins, quercetin glycosides), red wine and dealcoholized red wine (anthocyanins and resveratrol) and mix (poly)phenols (isoflavones, daidzein, genistein, anthocyanins, flavan-3-ols and procyanidins)	~60–1315	14–365	~6–39	(−17.0, +12.0)	No	No
Insulin (µIU/mL) (*n* = 10) [27,33,34,36,37,38,42,43,44,45]				14–365	~11–>100	(−3.3, +2.2)	No	No
HOMA- IR (*n* = 6) [27,33,37,42,43,45]				42–365	~27–>100	(−0.82, +0.24)	No	No

*n,* number of studies examined; CV%, calculated for the different groups examined. ^1^: Yes = all results in the same direction; No = results indicate increase and decrease; ^2^: Yes = all results reported to be significant (S) or not-significant (NS); No = some results were significant and some were not; MetS, metabolic syndrome; HOMA-IR, Homeostatic Model Assessment of Insulin Resistance.

**Table 4:** Summary of the collected responses of the lipid biomarkers to the daily consumption of mix (poly)phenols of different sources in postmenopausal women (for specific results in each study please refer to Tables S4–S7).

Lipid Profile	Study Characteristics				Effect (Change)			
	Type of Population	Source of (poly)phenols /Type of (poly)phenols	Doses (mg/day)	Duration (days)	Variability of the Results (CV%)	Effect Size Range	Consistency of the Change ^1^	Consistency of the Statistical Significance ^2^
T-C (mg/dL) (*n* = 15) [25,27,28,32,34,35,36,37,38,39,40,41,42,44,45]	Mix (healthy, osteopenia, dyslipidaemia)	Snack bar (soy protein, mix isoflavones), dried apple (proanthocyanins, hydroxycinnamic and anthocyanins), dried prunes (chlorogenic, neochlorogenic acids), chocolate 99% (procyanidins, epicatechins, quercetin glycosides), red wine and dealcoholized red wine (anthocyanins and resveratrol), beer and dealcoholized beer (prenylflavonoids) and mix (poly)phenols (isoflavones, daidzein, genistein, procyanidin, flavones, resveratrol, anthocyanins, quercetin, myricetin, kaempferol)	~43–1000	14–365	~10–48	(−22.0, +11.6)	No	No
LDL-C (mg/dL) (*n* = 15) [25,27,28,32,34,35,36,37,38,39,40,41,42,44,45]	Mix (healthy, osteopenia, dyslipidaemia)	Snack bar (soy protein, mix isoflavones), dried apple (proanthocyanins, hydroxycinnamic and anthocyanins), dried prunes (chlorogenic, neochlorogenic acids), chocolate 99% (procyanidins, epicatechins, quercetin glycosides), red wine and dealcoholized red wine (anthocyanins and resveratrol), beer and dealcoholized beer (prenylflavonoids) and mix (poly)phenols (isoflavones, daidzein, genistein, procyanidin, flavones, resveratrol, anthocyanins, quercetin, myricetin, kaempferol)	~43–1000	14–365	~13–92	(−23.9, +18.1)	No	No
HDL-C (mg/dL) (*n* = 16) [25,27,28,31,32,34,35,36,37,38,39,40,41,42,44,45]	Mix (healthy, osteopenia, dyslipidaemia, metabolic syndrom)	Snack bar (soy protein, mix isoflavones), dried apple (proanthocyanins, hydroxycinnamic and anthocyanins), dried prunes (chlorogenic, neochlorogenic acids), chocolate 99% (procyanidins, epicatechins, quercetin glycosides), red wine and dealcoholized red wine (anthocyanins and resveratrol), beer and dealcoholized beer (prenylflavonoids) and mix (poly)phenols (isoflavones, daidzein, genistein, procyanidin, flavones, resveratrol, anthocyanins, quercetin, myricetin, kaempferol)	~43–1000	14–365	~10–66	(−11.9, +11.1)	No	No
TGs (mg/dL) (*n* = 16) [25,27,28,31,32,34,35,36,37,38,39,40,41,42,44,45]	Mix (healthy, osteopenia, dyslipidaemia, metabolic syndrom)	Snack bar (soy protein, mix isoflavones), dried apple (proanthocyanidins, hydroxycinnamics and anthocyanins), dried prunes (chlorogenic, neochlorogenic acids), chocolate 99% (procyanidins, epicatechins, quercetin glycosides), red wine and dealcoholized red wine (anthocyanins and resveratrol), beer and dealcoholized beer (prenylflavonoids)	~43–1000	14–365	~10–80	(−23.0 +13.0)	No	No
		and mix (poly)phenols (isoflavones, daidzein, genistein, procyanidin, flavones, resveratrol, anthocyanins, quercetin, myricetin, kaempferol)						

*n*, number of studies examined; T-C, total cholesterol; LDL-C, low-density lipoprotein-cholesterol; HDL-C, high-density lipoprotein-cholesterol; TGs, triglycerides; CV%, calculated for the reported results in the different groups examined. ^1^: Yes = all results in the same direction; No = results indicate increase and decrease; ^2^: Yes = all results reported to be significant (S) or not-significant (NS); No = some results are significant some are not.

**Table 5:** Summary of the collected responses of blood pressure to the daily consumption of mix (poly)phenols of different sources in postmenopausal women (for specific results in each study go to Table S8).

Blood Pressure (Units)	Study Characteristics				Effect (Change)			
	Type of Population	Source of (poly)phenols/ Type of (poly)phenols	Doses (mg/day)	Duration (days)	Variability of the Results (CV%)	Effect Size Range	Consistency of the Change ^1^	Consistency of the Statistical Significance ^2^
SBP (mmHg) (*n* = 12) [25,26,29,31,32,34,35,37,39,42,44,45]	Mix (healthy, metabolic syndrome, dyslipidaemia, hypertensive)	Snack bar (soy protein, mix isoflavones), dried prunes (chlorogenic, neochlorogenic acids), chocolate 99% (procyanidins, epicatechins, quercetin glycosides), beer and dealcoholized beer (prenylflavonoids), red wine and dealcoholized red wine (anthocyanins and resveratrol) and mix (poly)phenols (isoflavones, daidzein, genistein, procyanidin, flavones, resveratrol, anthocyanins, or flavanols)	~43–1000	14–365	~7–29%	(−11.0, +16.0)	No	No
DBP (mmHg) (*n* = 12) [25,26,29,31,32,34,35,37,39,42,44,45]					~8–19%	(−7.0, +5.0)	No	No

*n*, number of studies examined; SBP, systolic blood pressure; DBP, diastolic blood pressure; CV %, calculated for the reported results in the different groups examined. ^1^: Yes = all results in the same direction; No = results indicate increase and decrease; ^2^: Yes = all results reported to be significant (S) or not-significant (NS); No = some results are significant some are not.

**Table 6:** Summary of the collected responses of the inflammatory biomarkers and oxidative biomarkers to the daily consumption of mix (poly)phenols of different sources in postmenopausal women (for specific results in each study, please refer to Tables S9 and S10).

Biomarkers (Units)	Study Characteristics				Effect (Change)			
	Type of Population	Source of (poly)phenols/ Type of (poly)phenols	Doses (mg/day)	Duration (days)	Variability of the Results (CV%)	Effect Size Range	Consistency of the Change ^1^	Consistency of the Statistical Significance ^2^
Inflammatory and cell adhesion biomarkers								
TNF-α (pg/L) (*n* = 3) [30,39,40]	Mix (healthy, hypertensive)	Blueberry powder (anthocyanins), lyophilized grape powder (anthocyanins, quercetin, myricetin, kaempferol and resveratrol) and elderberry extracts capsules (mix anthocyanins, mostly cyanidin-3-glucoside)	~200–845	28–84	~20–90%	(−3.0, +0.11)	No	No
IL-6 (pg/L) (*n* = 4) [28,39,40,44]	Mix (healthy, osteopenia)	Lyophilized grape powder (anthocyanins, quercetin, myricetin, kaempferol and resveratrol), elderberry extracts capsules (mix anthocyanins, mostly cyanidin-3-glucoside), olive leaf extract (mix (poly)phenols, >40% oleuropein) and anthocyanins capsules	~100–500	28–365	~25–>100%	(−0.50, +0.50)	No	Yes (NS)
CRP (mg/L) (*n* = 8) [28,29,30,37,39,40,41,44]	Mix (healthy, hypertensive, osteopenia)	Blueberry powder (anthocyanins), lyophilized grape powder (anthocyanins, quercetin, myricetin, kaempferol and resveratrol), elderberry extracts capsules (mix anthocyanins, mostly cyanidin-3- glucoside), olive leaf extract (mix (poly)phenols, >40% oleuropein), dried prunes (chlorogenic, neochlorogenic acids), dried apple (mix (poly)phenols), dried plums (mix (poly)phenols), snack bar (soy protein, mix isoflavones), and anthocyanins capsules	~66–845	28–365	~40–>100%	(−0.50, +1.0)	No	Yes (NS)
Adiponectin (µg/mL) (*n* = 3) [31,33,38]	Mix (healthy, hypertensive, overweight/ obese)	Green tea extract capsules (mostly EGCG plus EC, EGC, ECG, and GCG), cocoa (poly)phenols, soy isoflavones and catechins	~100–1300	60–365	~20–50%	(−1.0, +5.0)	No	No
sVCAM-1 (ng/mL) (*n* = 2) [25,44]	Mix (overweight and dyslipidaemia)	Anthocyanins capsules and cocoa beverage (mix flavanols)	~240–400	42–240	~20–40%	(−113.0, +16.0)	No	No
sICAM-1 (ng/mL) (*n* = 2) [25,44]	Mix (overweight and dyslipidaemia)	Anthocyanins capsules and cocoa beverage (mix flavanols)	~240–400	42–240	~15–55%	(−6.0, +29.0)	No	No
sP-Selectin (ng/mL) (*n* = 1) [25]	Dyslipidaemia	Cocoa beverage (mix flavanols)	~400	42	~30–55%	(+3.0)	NA	NA
sE-Selectin (ng/mL) (*n* = 1) [25]	Dyslipidaemia	Cocoa beverage (mix flavanols)	~400	42	~40–45%	(−5.0)	NA	NA
Oxidative stress biomarkers								
Ox-LDL (ng/mL)	Pre- and 1-stage hypertension	Blueberry powder (mix (poly)phenols and anthocyanins)	845	28, 56	~5–32%	(+32.4, +59.5) ^Ῡ^	Yes	NA
Isoprostanes (pg/mL serum)					~39–92%	(−3.6, +2.5) ^Ῡ^	No	NA
TBARS (µM)					~21–68%	(+0.30, +0.40) ^Ῡ^	Yes	NA
8-OHdG (ng/mL)					~13–26%	(−0.08, −0.04) ^Ῡ^	Yes	Yes
GSR (nmol/min/mL)					~24–>100%	(−1.4, +−0.10) ^Ῡ^	No	NA
GPx (nmol/min/mL)					~8–100%	(+3.1, +15.1) ^Ῡ^	Yes	NA
SOD (U/mL) (*n* = 1) [30]					~70–>100%	(−0.02, +0.03) ^Ῡ^	No	NA
Isoprostanes (ng/mg creatinine, urine) (*n* = 1) [30]	Healthy	Lyophilized grape powder (flavanols, anthocyanins, quercetin, myricetin, kaempferol, and resveratrol)	~210	28	~75–95%	NA	NA	NA
LPO (µM) (*n* = 1) [41]	Healthy	Dried plum or dried apple (mix (poly)phenols)	NI	90, 365	~42–>100%	(−7.7, +0.20) ^Ῡ^	No	NA

*n*, number of studies; TNF-α, tumor necrosis factor-alpha; IL-6, interleukin-6; CRP, C reactive protein; sVCAM-1, soluble vascular cell adhesion protein 1; sICAM-1, soluble intercellular adhesion molecule 1; Ox-LDL, oxidized LDL; 8-OHGdG, 8-hydroxy-guanosine; GSR, glutathione S-reductase; GPx, glutathione peroxidase; SOD, superoxide dismutase; TBARS, thiobarbituric acid reactive substances; LPO, lipid hydroperoxide; CV%, coefficient of variation calculated for the reported results in the different groups examined; EGCG, epigallocatechin gallate; EC, epicatechin; EGC, epigallocatechin; ECG, epicatechingallate; GCG, gallocatechin gallate; ^1^: Yes = all results in the same direction; No = results indicate either increase or decrease; ^2^: Yes = all results reported to be significant (S) or not-significant (NS); No = some results are (S) some are (NS); NA: not applicable; NI: not indicated; ^Ῡ^: a single trial that analyzes the variables under study in two different periods of time (28 d and 56 d).

## Supplementary Materials

The following are available online at https://www.mdpi.com/article/10.3390/nu13124276/s1. Table S1: PRISMA 2020 checklist item; Table S2: Risk of Bias Analysis; Table S3: Changes in biomarkers of glucose metabolism reported in human studies (parallel or crossover design) looking at the chronic effects of (poly)phenol-containing products in postmenopausal women without HRT; Table S4: Changes in total cholesterol reported in human studies (parallel or crossover design) looking at the chronic effects of (poly)phenol-containing products in postmenopausal women without HRT; Table S5: Changes in LDL-cholesterol reported in human studies (parallel or crossover design) looking at the effects of (poly)phenol-containing products in postmenopausal women without HRT; Table S6: Changes in HDL reported in human studies (parallel or crossover design) looking at the chronic effects of (poly)phenol-containing products in postmenopausal women without HRT; Table S7: Changes in total triglycerides (TGs) reported in human studies (parallel or crossover design) looking at the chronic effects of (poly)phenol-containing products in postmenopausal women without HRT; Table S8: Changes in systolic and diastolic blood pressure (SBP, DBP) reported in human studies (parallel or crossover design) looking at the chronic effects of (poly)phenol-containing products in postmenopausal women without HRT; Table S9: Changes in inflammatory and endothelial function biomarkers as reported in human studies (parallel or crossover design) looking at the chronic effects of (poly)phenol-containing products in postmenopausal women without HRT; Table S10: Changes in oxidative stress biomarkers reported in human studies (parallel or crossover design) looking at the chronic effects of (poly)phenol-containing products in postmenopausal women without HRT.
