# Supplementary material for: A Systematic Review of the Cardiometabolic Benefits of Plant Products Containing Mixed Phenolics and Polyphenols in Postmenopausal Women: Insufficient Evidence for Recommendations to This Specific Population
Source: Nutrients. 2021 Nov 27;13(12):4276. doi: 10.3390/nu13124276 (PMC8707028; doi:10.3390/nu13124276)
Supplement: Supplementary file 1 [file nutrients-13-04276-s001.zip › nutrients-1456469-supplementary.pdf]

SUPPLEMENTARY MATERIALS

Table S1. PRISMA 2020 Checklist

Section and Topic	Item #	Checklist item	Location where item is reported
**TITLE**			
Title	1	Identify the report as a systematic review.	1
**ABSTRACT**			
Abstract	2	See the PRISMA 2020 for Abstracts checklist.	
**INTRODUCTION**			
Rationale	3	Describe the rationale for the review in the context of existing knowledge.	1, 2
Objectives	4	Provide an explicit statement of the objective(s) or question(s) the review addresses.	2, 3
**METHODS**			
Eligibility criteria	5	Specify the inclusion and exclusion criteria for the review and how studies were grouped for the syntheses.	3, 4
Information sources	6	Specify all databases, registers, websites, organisations, reference lists and other sources searched or consulted to identify studies. Specify the date when each source was last searched or consulted.	3
Search strategy	7	Present the full search strategies for all databases, registers and websites, including any filters and limits used.	3, 4
Selection process	8	Specify the methods used to decide whether a study met the inclusion criteria of the review, including how many reviewers screened each record and each report retrieved, whether they worked independently, and if applicable, details of automation tools used in the process.	4, 5
Data collection process	9	Specify the methods used to collect data from reports, including how many reviewers collected data from each report, whether they worked independently, any processes for obtaining or confirming data from study investigators, and if applicable, details of automation tools used in the process.	4, 5
Data items	10a	List and define all outcomes for which data were sought. Specify whether all results that were compatible with each outcome domain in each study were sought (e.g. for all measures, time points, analyses), and if not, the methods used to decide which results to collect.	3 - 6
	10b	List and define all other variables for which data were sought (e.g. participant and intervention characteristics, funding sources). Describe any assumptions made about any missing or unclear information.	
Study risk of bias assessment	11	Specify the methods used to assess risk of bias in the included studies, including details of the tool(s) used, how many reviewers assessed each study and whether they worked independently, and if applicable, details of automation tools used in the process.	4, 5
Effect measures	12	Specify for each outcome the effect measure(s) (e.g. risk ratio, mean difference) used in the synthesis or presentation of results.	4
Synthesis	13a	Describe the processes used to decide which studies were eligible for each synthesis (e.g. tabulating the study intervention characteristics	4, 5

Section and Topic	Item #	Checklist item	Location where item is reported
methods		and comparing against the planned groups for each synthesis (item #5)).	
	13b	Describe any methods required to prepare the data for presentation or synthesis, such as handling of missing summary statistics, or data conversions.	4
	13c	Describe any methods used to tabulate or visually display results of individual studies and syntheses.	10
	13d	Describe any methods used to synthesize results and provide a rationale for the choice(s). If meta-analysis was performed, describe the model(s), method(s) to identify the presence and extent of statistical heterogeneity, and software package(s) used.	10
	13e	Describe any methods used to explore possible causes of heterogeneity among study results (e.g. subgroup analysis, meta-regression).	
	13f	Describe any sensitivity analyses conducted to assess robustness of the synthesized results.	
Reporting bias assessment	14	Describe any methods used to assess risk of bias due to missing results in a synthesis (arising from reporting biases).	
Certainty assessment	15	Describe any methods used to assess certainty (or confidence) in the body of evidence for an outcome.	
**RESULTS**			
Study selection	16a	Describe the results of the search and selection process, from the number of records identified in the search to the number of studies included in the review, ideally using a flow diagram.	5, 6
	16b	Cite studies that might appear to meet the inclusion criteria, but which were excluded, and explain why they were excluded.	5, 6
Study characteristics	17	Cite each included study and present its characteristics.	6, Table 2
Risk of bias in studies	18	Present assessments of risk of bias for each included study.	Supplementary Material (Table S2)
Results of individual studies	19	For all outcomes, present, for each study: (a) summary statistics for each group (where appropriate) and (b) an effect estimate and its precision (e.g. confidence/credible interval), ideally using structured tables or plots.	Supplementary Material (Tables S3 to S10) Tables 3 to 6
Results of syntheses	20a	For each synthesis, briefly summarise the characteristics and risk of bias among contributing studies.	6, Table 2

Section and Topic	Item #	Checklist item	Location where item is reported
	20b	Present results of all statistical syntheses conducted. If meta-analysis was done, present for each the summary estimate and its precision (e.g. confidence/credible interval) and measures of statistical heterogeneity. If comparing groups, describe the direction of the effect.	10, 12, 15 Tables 3 to 6
	20c	Present results of all investigations of possible causes of heterogeneity among study results.	
	20d	Present results of all sensitivity analyses conducted to assess the robustness of the synthesized results.	
Reporting biases	21	Present assessments of risk of bias due to missing results (arising from reporting biases) for each synthesis assessed.	
Certainty of evidence	22	Present assessments of certainty (or confidence) in the body of evidence for each outcome assessed.	Tables 3 to 6
**DISCUSSION**			
Discussion	23a	Provide a general interpretation of the results in the context of other evidence.	
	23b	Discuss any limitations of the evidence included in the review.	18 - 22
	23c	Discuss any limitations of the review processes used.	18 - 22
	23d	Discuss implications of the results for practice, policy, and future research.	18 - 22
**OTHER INFORMATION**			
Registration and protocol	24a	Provide registration information for the review, including register name and registration number, or state that the review was not registered.	
	24b	Indicate where the review protocol can be accessed, or state that a protocol was not prepared.	3, 4
	24c	Describe and explain any amendments to information provided at registration or in the protocol.	
Support	25	Describe sources of financial or non-financial support for the review, and the role of the funders or sponsors in the review.	23
Competing interests	26	Declare any competing interests of review authors.	23
Availability of data, code and other materials	27	Report which of the following are publicly available and where they can be found: template data collection forms; data extracted from included studies; data used for all analyses; analytic code; any other materials used in the review.	Supplementary Material (Tables S1 to S10)

From: Page MJ, McKenzie JE, Bossuyt PM, Boutron I, Hoffmann TC, Mulrow CD, et al. The PRISMA 2020 statement: an updated guideline for reporting systematic reviews. BMJ 2021;372:n71. doi: 10.1136/bmj.n71.

Table S2. Risk of Bias Tools.

Reference	Selection bias	Performance bias—blinding		Attrition bias – flow of participants	Other bias			Risk of bias Score (Overall)*
		Blinding	Performance		Baseline comparability	Data report	Funding	
Cheng et al., 2004 [36]	1	2	0	1	1	1	1	7.0
Sathyapalan et al., 2018 [37]	1	2	0	1	1	1	1	7.0
Myasoedova et al., 2016 [35]	0	2	0	1	1	1	1	6.0
Wu et al., 2012 [38]	0	2	0	0	1	1	1	5.0
Curtis et al., 2009 [39]	0	2	0.5	1	0	1	1	5.5
Zern et al., 2005 [40]	0	1	1	0	0	1	1	4.0
Chai et al., 2012 [41]	1	1	0.5	1	1	1	1	6.5
Al-Dashti et al., 2019 [34]	1	0	0.5	1	0	1	1	4.5
García-Yu et al., 2020 [42]	1	1	0.5	1	1	1	1	6.5
García-Yu et al., 2021[43]	1	1	0.5	1	1	1	1	6.5
Estévez-Santiago et al., 2019 [44]	0	0	0	1	1	1	1	4.0
Trius-Soler et al., 2021 [32]	0	0	0.5	1	1	1	1	4.5

**Filip et al., 2015 [28]**	**1**	**2**	**0.5**	**1**	**1**	**1**	**1**	**7.5**
**Wang-Polagruto et al., 2006 [25]**	**0**	**2**	**0.5**	**0**	**1**	**1**	**1**	**5.5**
**Naissides et al., 2006a [26]**	**1**	**0**	**1**	**1**	**1**	**1**	**1**	**6.0**
**Naissides et al., 2006b [27]**	**1**	**0**	**1**	**0**	**1**	**1**	**1**	**5.0**
**Aubertin-Leheudre et al., 2008 [45]**	**0**	**2**	**0.5**	**1**	**0**	**1**	**1**	**5.5**
**Dostal et al., 2016 [33]**	**1**	**2**	**0.5**	**1**	**1**	**1**	**1**	**7.5**
**Johnson et al., 2015 [29]**	**1**	**2**	**0**	**1**	**1**	**1**	**1**	**7.0**
**Johnson et al., 2017 [30]**	**1**	**2**	**0.5**	**1**	**1**	**1**	**1**	**7.5**
**D´Anna et al., 2014 [31]**	**1**	**0**	**0.5**	**1**	**1**	**1**	**1**	**5.5**

*: Risk of bias Score: low risk (≥8 and ≤10), moderate risk (≥5 and <8), high risk (<5).

Table S3. Changes in markers of glucose metabolism as reported in human RCTs (parallel or crossover design) looking at the chronic effects of (poly)phenol-containing products in postmenopausal women, without hormone replacement therapy.

**References**	**Health status**	**Treatment Groups (*N* participants per group)**	**(Poly) phenols**	**Dose (poly) phenols (mg /d)**	**Duration (d)**	**Markers of glucose metabolism**					
						**Parameter**	**Control/Placebo/ Comparator**	**Δ (A – B) (*p*-value)**	**Treatment**	**Δ (A – B) (*p*-value)**	**Δ (Treatment – Control/Placebo) (*p*-value)**
**Cheng et al., 2004 [36]**	Healthy	Capsules<br><br>Estrogen (*11*)<br>Isoflavone (*17*)	Isoflavones (daidzein and genistein) in evening primrose oil	100	90<br>180	**Glucose** (mg/dL)	B: 118.0 ± 46.0<br>A90: 94.0 ± 20.0<br>A180: 92.0 ± 21.0	**-24.0**<br>**-26.0**<br>(0.001)	B: 102.0 ± 18.0<br>A90: 90.0 ± 24.0<br>A180: 81.0 ± 25.0	**-12.0**<br>**-21.0**<br>(0.001)	**+12.0**<br>**+5.0**<br>(NS)
						**Insulin** (µIU/mL)	B: 11.0 ± 9.0<br>A90: 6.0 ± 7.0<br>A180: 6.0 ± 11.0	**-5.0**<br>**-5.0**<br>(0.005)	B: 12.0 ± 12.0<br>A90: 4.0 ± 3.0<br>A180: 5.0 ± 8.0	**-8.0**<br>**-7.0**<br>(0.005)	**-3.0**<br>**-2.0**<br>(NS)
**Sathyapalan et al., 2018 [37]**	Healthy	Snack bars (soy protein free isoflavones or with isoflavones)<br><br>Placebo (*60*)<br>Treated (*60*)	Mix isoflavones	66	180	**Glucose** (mg/dL)	B: 91.8 ± 28.8<br>A: 90.0 ± 16.2	-1.8<br>(NI)	B: 93.6 ± 12.6<br>A: 79.2 ± 9.0	-14.4<br>(NI)	-12.6<br>(<0.010)
						**Insulin** (µIU/mL)	B: 5.7 ± 3.7<br>A: 5.8 ± 3.7	+0.11<br>(NI)	B: 5.8 ± 3.6<br>A: 2.6 ± 1.9	-3.1<br>(NI)	-3.3<br>(<0.010)
						**HOMA-IR**	B: 1.4 ± 1.8<br>A: 1.4 ± 1.4	-0.04<br>(NI)	B: 1.4 ± 1.0<br>A: 0.50 ± 0.40	-0.86<br>(NI)	-0.82<br>(<0.010)
**Wu et al., 2012 [38]**	Healthy	Green tea extract capsules<br><br>Placebo (*32*)<br>T_1: G400 (*37*)<br>T_2: G800 (*34*)	Mix (poly)phenols (mostly EGCG plus EC, EGC, ECG, and GCG)	T_1: 400<br>T_2: 800	60	**Glucose** (mg/dL)	B: 97.0 [91.0, 103.0]$^{\delta}$<br>A: 99.0 [94.0, 105.0]$^{\delta}$	**+2.0**<br>(0.052)	**T_1: Group G400**<br>B: 99.0 [94.0, 105.0]$^{\delta}$<br>A: 98.0 [93.0, 103.0]$^{\delta}$<br><br>**T_2: Group G800**<br>B: 103.0 [97.0, 110.0]$^{\delta}$<br>A: 101.0 [95.0, 106.0]$^{\delta}$	**G400**<br>**-1.0**<br>(NS)<br><br>**G800**<br>**-2.0**<br>(NS)	**Group G400 *vs* P**<br>**-3.0**<br>(NI)<br><br>**Group G800 *vs* P**<br>**-4.0**<br>(NI)<br><br>**Groups *vs* P**<br>NI<br>(0.008)<br><br>**Group G800 *vs* G400**<br>**-1.0**<br>(NS)

						**Insulin** (μIU/mL)	B: 7.3 [5.7, 9.5]$^{\delta}$<br>A: 8.9 [6.9, 11.5]$^{\delta}$	**+1.6**<br>(0.059)	**T_1: Group G400**<br>B: 8.5 [6.6, 10.9]$^{\delta}$<br>A: 8.3 [6.5, 10.9]$^{\delta}$<br><br>**T_2: Group G800**<br>B: 9.5 [7.4, 12.3]$^{\delta}$<br>A: 9.0 [7.0, 11.5]$^{\delta}$	**G400**<br>**-0.20**<br>(NS)<br><br>**G800**<br>**-0.50**<br>(NS)	**Group G400 *vs* P**<br>**-1.8**<br>(NI)<br><br>**Group G800 *vs* P**<br>**-2.1**<br>(NI)<br><br>**Groups *vs* P**<br>NI<br>(0.010)<br><br>**Group G800 *vs* G400**<br>**-0.30**<br>(NS)
**Curtis et al., 2009 [39]**	Healthy	Elderberry extract capsules (125 mg)<br><br>Placebo (*26*)<br>Treated (*26*)	Mix anthocyanins (mostly cyanidin-3-glucoside) per capsule	500	84	**Glucose** (mg/dL)	B: 86.4 ± 9.0<br>A: 90.0 ± 10.8	**+3.6**<br>(NS)	B: 88.2 ± 7.2<br>A: 88.2 ± 7.2	**0.0**<br>(NS)	**-3.6**<br>(NS)
**Al-Dashi et., al 2019 [34]**	Healthy	Prunes<br><br>T_1: Low dose 14 g (*27*)<br>T_2: High dose 42 g (*27*)	Mix (poly) phenols (chlorogenic, neochlorogenic acids, others)	NI	14	**Glucose** (mg/dL)	**T_1: Low dose**<br>B: 95.0$^{\delta}$ ± 16.0<br>A: 96.0$^{\delta}$ ± 15.0	**+3.0**<br>(0.030)	**T_2: High dose**<br>B: 98.0$^{\delta}$ ± 14.0<br>A: 96.0$^{\delta}$ ± 15.0	**-3.0**<br>(NS)	**High *vs* Low**<br>**-5.0**<br>(NS)
						**Insulin** (μIU/mL)	**T_1: Low dose**<br>B: 4.5$^{\delta}$ ± 3.9<br>A: 4.6$^{\delta}$ ± 3.6	**+0.15**<br>(NS)	**T_2: High dose**<br>B: 4.4$^{\delta}$ ± 3.9<br>A: 4.5$^{\delta}$ ± 3.9	**+0.10**<br>(NS)	**High *vs* Low**<br>**-0.05**<br>(NS)
**García-Yu et al., 2020 [42]**	Healthy	10g of dark chocolate (99% cocoa)<br><br>Control (*66*)<br>Treated (*71*)	Flavanols	65	180	**Glucose** (mg/dL)	B: 86.2 ± 8.5<br>A: 87.2 ± 8.7	+1.0<br>(NS)	B: 86.4 ± 8.8<br>A: 86.6 ± 10.0	+0.20<br>(NS)	-0.80<br>(NS)
						**Insulin*** (mg/dL)	B: 7.5 ± 2.9<br>A: 7.8 ± 3.6	+0.30<br>(NS)	B: 8.2 ± 3.4<br>A: 8.3 ± 5.1	+0.10<br>(NS)	-0.19<br>(NS)
						**HOMA-IR**	B: 1.6 ± 0.70<br>A: 1.7 ± 0.90	+0.10<br>(NS)	B: 1.8 ± 0.80<br>A: 1.8 ± 1.4	+0.10<br>(NS)	-0.02<br>(NS)
	Healthy	10g of dark chocolate (99% cocoa)	Flavanols	65	120	**Insulin*** (mg/dL)	B: 7.3 ± 2.7<br>A: NI	NI<br>(NS)	B: 8.6 ± 3.4<br>A: NI	NI<br>(NS)	NI<br>(NS)

**García- Yu et al., 2021 [43]**		Control (61)<br>Treated (67)				**HOMA-IR**	B: 1.6 ± 0.60<br>A: NI	NI<br>(NS)	B: 1.9 ± 0.90<br>A: NI	NI<br>(NS)	NI<br>(NS)
**Estévez-Santiago et al., 2019 [44]**	Healthy	Capsules<br><br>T_1: Group X *(26)*<br>T_2: Group A *(23)*<br>T_3: Group A+X *(23)*	T_1: Anthocyanins<br>T_2: Xanthophylls (lutein + zeaxanthin)<br>T_3: Anthocyanins and xanthophylls (lutein + zeaxanthin)	T_1: 8.0<br>T_2: 60<br>T_3: 68	120<br>240	**Glucose**<br>(mg/dL)	**T_1: Group X**<br>B: 92.3 ± 7.4<br>A120: 92.2 ± 6.7<br>A240: 91.3 ± 10.1	 **-0.18**<br>**-1.1**<br>(NS)	**T_2: Group A**<br>B: 89.6 ± 8.5<br>A120: 92.2 ± 9.0<br>A240: 92.2 ± 6.7<br><br>**T_3: Group A+X**<br>B: 87.8 ± 6.8<br>A120: 89.6 ± 6.7<br>A240: 85.5 ± 5.9	 **-2.5**<br>**-2.5**<br>(NS)<br><br><br>**+1.8**<br>(NS)<br>**-2.3**<br>(< 0.050)	**Group A *vs* X**<br>**-2.3**<br>**-1.4**<br>(NS)<br><br>**Group A+X *vs* X**<br>**+1.9**<br>**-1.2**<br>(NS)<br><br>**Group A+X *vs* A**<br>**+4.3**<br>**+0.18**<br>(NS)
						**Insulin**<br>(µIU/mL)	**T_1: Group X**<br>B: 7.3 ± 4.3<br>A120: 7.6 ± 3.9<br>A240: 7.3 ± 3.6	 **+0.30**<br>**0.00**<br>(NS)	**T_2: Group A**<br>B: 7.1 ± 3.1<br>A120: 7.3 ± 3.8<br>A240: 8.0 ± 3.4<br><br>**T_3: Group A+X**<br>B: 5.9 ± 2.0<br>A120: 6.8 ± 3.4<br>A240: 6.6 ± 3.2	 **+0.20**<br>**+0.90**<br>(NS)<br><br><br>**+0.90**<br>**+0.70**<br>(NS)	**Group A *vs X***<br>**-0.10**<br>**+0.90**<br>(NS)<br><br>**Group A+X *vs* X**<br>**+0.60**<br>**+0.70**<br>(NS)<br><br>**Group A+X *vs* A**<br>**+0.70**<br>**-0.20**<br>(NS)
**Trius-Soler et al., 2021 [32]**	Healthy	Beer beverage with and without alcohol<br>Control *(14)*<br>T_1: Group AB *(16)*<br>T_2: Group NAB *(7)*	Prenylflavonoids	T_1: 0.359<br>T_2: 0.259	180	**Glucose**<br>(mg/dL)	A: 91.0 ± 6.0<br>B: NI	NI<br>(NS)	**T_1: Group AB**<br>B: 94.0 ± 8.0<br>A:NI<br><br>**T_2: Group NAB**<br>B: 97.0 ± 12.0<br>A: NI	 NI<br>(NS)<br><br><br>NI<br>(NS)	**Group AB *vs* C**<br>+0.70<br>(NS)<br><br>**Group NAB *vs* C**<br>+3.0<br>(NS)

											**Groups** ***vs*** **C**<br>NI<br>(NS)<br><br>**Group NAB** ***vs*** **AB**<br>**+2.0**<br>(NS)
**D´Anna et al., 2014 [31]**	Diagnosis of metabolic syndrome (Obese)	Capsules (30 mg of cocoa (poly)phenols, 80 mg of soy isoflavones, and 2 g of myo-inositol)<br><br>Placebo (*21*)<br>Treated (*22*)	Cocoa (poly)phenols and soy isoflavones	110	180	**Glucose** (mg/dL)	B: 105.0 ± 7.0<br>A: 108.0 ± 10.0	**+3.0**<br>(NS)	B: 110.0 ± 10.0<br>A: 96.0 ± 7.0	**-14.0**<br>(<0.001)	**-17.0**<br>(<0.001)
**Naissides et al., 2006b [27]**	Dyslipidaemia (high cholesterol)	Red wine RW (2.5 g (poly)phenols/L)<br><br>Control (400 mL water) (*16*)<br><br>T_1: Group DRW non-alcohol (400 mL) (*15*)<br><br>T_2: Group RW alcohol (400 mL) (*14*)	Mix wine (poly) phenols	1000	42	**Glucose** (mg/dL)	B: 91.98 ± 7.9<br>A: 91.98 ± 7.2	**0.0**<br>(NS)	**T_1: Group DRW**<br>B: 92.9 ± 7.6<br>A: 91.3 ± 4.1<br><br>**T_2: Group RW**<br>B: 91.1 ± 5.4<br>A: 94.3 ± 5.9	**DRW**<br>**-1.6**<br>(NS)<br><br>**RW**<br>**+3.2**<br>(NS)	**Group DRW** ***vs*** **C**<br>**-1.6**<br>(NS)<br><br>**Group RW** ***vs*** **C**<br>**+3.2**<br>(NS)<br><br>**Groups** ***vs*** **C**<br>NI<br>(NS)<br><br>**Group DRW** ***vs*** **RW**<br>**-4.9**<br>(NS)
						**Insulin** (µIU/mL)	B: 5.5 ± 3.5<br>A: 5.3 ± 0.60	**-0.16**<br>(NS)	**T_1: Group DRW**<br>B: 6.1 ± 3.1<br>A: 5.0 ± 1.5<br><br>**T_2: Group RW**<br>B: 5.4 ± 4.0<br>A: 5.8 ± 5.9	**DRW**<br>**-1.0**<br>(NS)<br><br>**RW**<br>**+0.40**<br>(NS)	**Group DRW** ***vs*** **C**<br>**-0.87**<br>(NS)<br><br>**Group RW** ***vs*** **C**<br>**+0.56**<br>(NS)

											**Groups *vs* C**<br>NI<br>(NS)<br><br>**Group DRW *vs* RW**<br>**-1.4**<br>(NS)
						**HOMA-IR**	B: 1.3 ± 0.88<br>A: 1.2 ± 0.56	**-0.05**<br>(NS)	**T₁: Group DRW**<br>B: 1.4 ± 0.73<br>A: 1.1 ± 0.34<br><br>**T₂: Group RW**<br>B: 1.2 ± 0.97<br>A: 1.4 ± 1.5	**DRW**<br>**-0.25**<br>(NS)<br><br>**RW**<br>**+0.13**<br>(NS)	**Group DRW *vs* C**<br>**-0.20**<br>(NS)<br><br>**Group RW *vs* C**<br>**+0.18**<br>(NS)<br><br>**Groups *vs* C**<br>NI<br>(NS)<br><br>**Group DRW *vs* RW**<br>**-0.38**<br>(NS)
**Aubertin-Leheudre et al., 2008 [45]**	Obese	Soybeans capsules (17.5 mg)<br><br>Placebo *(18)*<br>Treated *(21)*	Mix isoflavones (daidzein, glycitein, and genistein)	70	180	**Glucose** (mg/dL)	B: 89.3 ± 10.6<br>A: 89.5 ± 7.4	**+0.18**<br>(NS)	B: 90.2 ± 8.8<br>A: 88.4 ± 8.6	**-1.8**<br>(NS)	**-1.9**<br>(NS)
						**Insulin** (µIU/mL)	B: 12.0 ± 4.6<br>A: 8.4 ± 3.8	**-3.6**<br>(NS)	B: 8.8 ± 3.4<br>A: 7.4 ± 2.8	**-1.4**<br>(NS)	**+2.2**<br>(NS)
						**HOMA-IR**	B: 1.4 ± 0.61<br>A: 1.1 ± 0.76	**-0.26**<br>(NS)	B: 1.0 ± 0.45<br>A: 0.98 ± 0.78	**-0.02**<br>(NS)	**+0.24**<br>(NS)
**Dostal et al., 2016 [33]**	Obese and overweight	Capsules (1315 mg of catechins)<br><br>Placebo *(120)*<br>Treated *(117)*	Catechins	1315	365	**Glucose** (mg/dL)	B: 97.3 [95.0, 99.7]$^{\delta}$<br>A: 94.7 [92.5, 97.0]$^{\delta}$	-1.9<br>(NS)	B: 97.6 [95.3, 100.0]$^{\delta}$<br>A: 95.3 [93.0, 97.6]$^{\delta}$	-1.6<br>(NS)	**+0.37**<br>(NS)
						**Insulin** (µIU/mL)	B: 6.2 [5.7, 6.9]$^{\delta}$<br>A: 6.6 [6.0, 7.3]$^{\delta}$	+0.29<br>(NS)	B: 6.7 [6.05, 7.31]$^{\delta}$<br>A: 6.9 [6.33, 7.65]$^{\delta}$	+0.31<br>(NS)	**+0.02**<br>(NS)
						**HOMA-IR**	B: 1.5 [1.4, 1.7]$^{\delta}$<br>A: 1.6 [1.4, 1.7]$^{\delta}$	+0.05<br>(NS)	B: 1.6 [1.5, 1.8]$^{\delta}$<br>A: 1.6 [1.5, 1.8]$^{\delta}$	+0.07<br>(NS)	**+0.02**<br>(NS)

Data are presented as the mean ± SD unless otherwise stated; HOMA-IR: Homeostatic Model Assessment of insulin resistance; $^{\delta}$: geometric mean [95% Confidence intervals]; B: Before intervention; A: After intervention; NS: Not significant; NI: Not indicated; C: Control; T: Treated; P: Placebo; EGCG: Epigallocatechin gallate; EC: Epicatechin; EGC: Epigallocatechin; ECG: Epicatechin gallate; GCG: Gallocatechin gallate; AB: Alcoholic beer; NAB: Dealcoholized beer; DRW: Dealcoholized red wine; RW: Red wine; **numbers in bold** are the changes estimated as the mean difference: (A) after – (B) before (intra-group) or treated – control or placebo or comparator (between groups); *The units used by authors were not the most common ones and the transformation to conventional or standardized units (µIU/mL) resulted in incongruent results.

Table S4. Changes in total cholesterol as reported in human RCTs (parallel or crossover design) looking at the chronic effects of (poly)phenol-containing products in postmenopausal women, without hormone replacement therapy or lipid-lowering therapy.

References	Health status	Treatment Groups (*N* participants per group)	(Poly)phenols	Dose (poly)phenols (mg /d)	Duration (d)	Total circulating cholesterol (mg/dL)				
						Control/Placebo/ Comparator	Δ (A – B) (*p*-value)	Treatment	Δ (A – B) (*p*-value)	Δ (Treatment – Control/Placebo) (*p*-value)
**Cheng et al., 2004 [36]**	Healthy	Capsules<br><br>Estrogen (*11*)<br>Isoflavone (*17*)	Isoflavones (daidzein and genistein) in evening primrose oil	100	90<br>180	B: 214.6 ± 27.4<br>A90: 225.8 ± 38.2<br>A180: 219.6 ± 36.6	**+11.2**<br>**+5.0**<br>(NS)	B: 197.2 ± 32.0<br>A90: 212.3 ± 32.8<br>A180: 207.6 ± 32.4	**+15.1**<br>**+10.4**<br>(NS)	**+3.9**<br>**+5.4**<br>(NS)
**Sathyapalan et al., 2018 [37]**	Healthy	Snack bars (soy protein free isoflavones or with isoflavones)<br><br>Placebo (*60*)<br>Treated (*60*)	Mix isoflavones	66	180	B: 223.9 ± 30.9<br>A: 220.0 ± 30.9	-3.9<br>(NS)	B: 223.9 ± 34.7<br>A: 223.9 ± 34.7	0.0<br>(NS)	+3.9<br>(NS)
**Myasoedova et al., 2016 [35]**	Healthy	Mixed herbs capsules (500 mg of mix grape seeds, green tea leaves, hop cone and garlic powder)<br><br>Placebo (*71*)<br>Treated ×3 capsules/d (*56*)	Mix (poly)phenols (procyanidin, genistein, daidzein, flavones, resveratrol, other phenolics)	283	365	B: 252.0 ± 42.0<br>A: NI	-13.0<br>(0.020)	B: 271.0 ± 55.0<br>A: NI	-17.0<br>(0.010)	**-4.0**<br>(NS)
**Wu et al., 2012 [38]**	Healthy	Green tea extract capsules (G)<br><br>Placebo (*32*)<br>T_1: Group G400 (*37*)<br>T_2: Group G800 (*34*)	Mix (poly)phenols (mostly EGCG plus EC, EGC, ECG, and GCG)	T_1: 400<br>T_2: 800	60	B: 216.0 [203.0, 231.0]$^{\delta}$<br>A: 216.0 [203.0, 229.0]$^{\delta}$	**0.0**<br>(NS)	**T_1: Group G400**<br>B: 218.0 [205.0, 231.0]$^{\delta}$<br>A: 207.0 [196.0, 219.0]$^{\delta}$<br><br>**T_2: Group G800**<br>B: 208.0 [195.0, 221.0]$^{\delta}$<br>A: 202.0 [190.0, 214.0]$^{\delta}$	**G400**<br>**-11.0**<br>(0.012)<br><br>**G800**<br>**-6.0**<br>(0.045)	**Group G400 vs P**<br>**-11.0**<br>(0.072)<br><br>**Group G800 *vs* P**<br>**-6.0**<br>(0.072)<br><br>**Groups *vs* P**<br>NI<br>(NS)<br><br>**Group G800 *vs* G400**<br>**+5.0**

										(NS)
**Curtis et al., 2009 [39]**	Healthy	Elderberry extract capsules (125 mg)<br><br>Placebo (*26*)<br>Treated (*26*)	Mix anthocyanins (mostly cyanidin-3-glucoside) per capsule	500	84	B: 212.3 ± 23.2<br>A: 204.6 ± 34.7	**-7.7**<br>(NS)	B: 208.4 ± 27.0<br>A: 212.3 ± 27.0	**+3.9**<br>(NS)	**+11.6**<br>(NS)
**Zern et al., 2005 [40]**	Healthy	Lyophilized grape powder<br><br>Placebo (*20*)<br>Treated (*20*)	Mix grape (poly)phenols (anthocyanins, quercetin, myricetin, kaempferol and resveratrol)	~210	28	B: 216.2 ± 38.6<br>A: 223.9 ± 42.5	**+7.7**<br>(NS)	B: 216.2 ± 38.6<br>A: 220.0 ± 42.5	**+3.9**<br>(NS)	**-3.9**<br>(NS)
**Chai et al., 2012 [41]**	Healthy	Dried plum or dried apple<br><br>T_1: Group DP (*55*)<br>T_2: Group DA (*45*)	Mix (poly)phenols	NI	90<br>180<br>365	**T_1: Group DP**<br>B: 189.5 ± 91.0<br>A90: 185.7 ± 82.0<br>A180: 185.7 ± 85.0<br>A365: 182.6 ± 85.0	 **-3.9**<br>**-3.9**<br>**-6.9**<br>(NS)	**T_2: Group DA**<br>B: 196.9 ± 70.0<br>A90: 176.8 ± 53.0<br>A180: 170.6 ± 56.0<br>A365: 169.5 ± 83.0	 **-20.1**<br>**-26.3**<br>**-27.4**<br>(0.002)	**Group DA *vs* DP**<br>**-16.2**<br>**-22.4**<br>**-20.5**<br>(365d)<br>(<0.050)
**Al-Dashi et al., 2019 [34]**	Healthy	Prunes<br><br>T_1: Low dose 14 g (*27*)<br>T_2: High dose 42 g (*27*)	Mix (poly)phenols (chlorogenic, neochlorogenic acids, others)	NI	14	**T_1: Low dose**<br>B: 213.0^{δ} ± 43.0<br>A: 208.0^{δ} ± 34.0	 **-5.0**<br>(NS)	**T_2: High dose**<br>B: 209.0^{δ} ± 37.0<br>A: 208.0^{δ} ± 33.0	 **-0.60**<br>(NS)	**High *vs* Low**<br>**+5.0**<br>(NS)
**García-Yu et al., 2020 [42]**	Healthy	10g of dark chocolate (99% cocoa)<br><br>Control (*66*)<br>Treated (*71*)	Flavanols	65	180	B: 204.0 ± 26.6<br>A: 205.0 ± 30.2	+0.90<br>(NS)	B: 211.0 ± 28.5<br>A: 212.0 ± 34.6	+1.3<br>(NS)	+0.44<br>(NS)
**Estévez-Santiago et al., 2019 [44]**	Healthy	Capsules<br><br>T_1: Group X *(26)*<br>T_2: Group A *(23)*<br>T_3: Group A+X *(23)*	T_1: Xanthophylls (lutein + zeaxanthin)<br>T_2: Anthocyanins<br>T_3: Anthocyanins and xanthophylls (lutein + zeaxanthin)	T_1: 8.0<br>T_2: 60<br>T_3: 68	120<br>240	**T_1: Group X**<br>B: 210.4 ± 26.6<br>A120: NI<br>A240: NI	 NI<br>NI<br>(NS)	**T_2: Group A**<br>B: 208.8 ± 38.6<br>A120: NI<br>A240: NI<br><br>**T_3: Group A+X**<br>B: 221.9 ± 22.4<br>A120: NI<br>A240: NI	 NI<br>NI<br>(NS)<br><br><br>NI<br>NI<br>(NS)	**Group A *vs* X**<br>NI<br>NI<br>(NS)<br><br>**Group A+X *vs* X**<br>NI<br>NI<br>(NS)

										**Group A+X *vs* A**<br>NI<br>NI<br>(NS)
**Trius-Soler et al., 2021 [32]**	Healthy	Beer beverage with anc without alcohol<br>Control (*14*)<br>T_1: Group AB (*16*)<br>T_2: Group NAB (*7*)	Prenylflavonoids	T_1: 0.359<br>T_2: 0.259	180	B: 185.0 ± 30.0<br>A: NI	NI<br>(NS)	**T_1: Group AB**<br>B: 206.0 ± 22.0<br>A: NI<br><br>**T_2: Group NAB**<br>B: 208.0 ± 27.0<br>A: NI	NI<br>(NS)<br><br>NI<br>(NS)	**Group AB *vs* C**<br>-6.0<br>(NS)<br><br>**Group NAB *vs* C**<br>-10.0<br>(NS)<br><br>**Groups *vs* C**<br>NI<br>(NS)<br><br>**Group NAB *vs* AB**<br>-4.0<br>(NS)
**Wang-Polagruto et al., 2006 [25]**	Dyslipidaemia (high cholesterol)	Flavanol cocoa beverage<br><br>T_1: Low flavanol (*16*)<br>T_2: High flavanol (*16*)	Mix flavanols	T_1: 43<br>T_2: 446	42	**T_1: Low flavanol**<br>B: 240.0 ± 32.0<br>A: NI	NI<br>(NS)	**T_2: High flavanol**<br>B: 235.0 ± 32.0<br>A: NI	NI<br>(NS)	**High *vs* Low**<br>NI<br>(NS)
**Naissides et al., 2006b [27]**	Dyslipidaemia (high cholesterol)	Red wine RW (2.5 g (poly)phenols/L)<br><br>Control (400 mL water) (*16*)<br><br>T_1: Group DRW alcohol (400 mL) (*15*)<br><br>T_2: Group RW non-alcohol (400 mL) (*14*)	Mix (poly)phenols	1000	42	B: 238.2 ± 23.0<br>A: 242.8 ± 23.0	**+4.6**<br>(NS)	**T_1: Group DRW**<br>B: 242.0 ± 39.0<br>A: 238.9 ± 48.0<br><br>**T_2: Group RW**<br>B: 241.6 ± 20.0<br>A: 241.3 ± 25.0	**-3.1**<br>(NS)<br><br>**-0.38**<br>(NS)	**Group DRW *vs* C**<br>**-7.7**<br>(NS)<br><br>**Group RW *vs* C**<br>**-5.0**<br>(NS)<br><br>**Groups *vs* C**<br>NI<br>(NS)<br><br>**Group DRW *vs* RW**<br>**-2.7**<br>(NS)

**Aubertin-Leheudre et al., 2008 [45]**	Obese	Soybeans capsules (17.5 mg)<br><br>Placebo (*18*)<br>Treated (*21*)	Mix isoflavones (daidzein, glycitein, and genistein)	70	180	B: 205.7 ± 32.0<br>A: 218.5 ± 28.2	**+12.7**<br>(NS)	B: 208.8 ± 33.9<br>A: 201.9 ± 27.8	**-6.9**<br>(NS)	**-19.7**<br>(NS)
**Filip et al., 2015 [28]**	Osteopenia (mix cholesterol levels)	Olive leaf extract<br><br>Placebo (400 mg Ca capsules) (*21*)<br>Treated (400 mg Ca + 250 mg olive extract capsules) (*27*)	Mix (poly)phenols (>40% oleuropein)	>100 oleuropein	180<br>365	B: 249.1 ± 35.1<br>A180: 220.7 ± 32.5<br>A365: 233.4 ± 32.6	-28.4<br>-15.7<br>(365d)<br>(0.010)	B: 236.2 ± 51.9<br>A180: 219.0 ± 41.3<br>A365: 209.9 ± 35.5	-17.2<br>-26.2<br>(365d)<br>(0.010)	+11.2<br>-10.5<br>(NS)

Data are presented as the mean ± SD unless otherwise stated; B: Before intervention; A: After intervention; NS: Not significant: NI: Not indicated; C: Control; T: Treated; P: Placebo; EGCG: Epigallocatechin gallate; EC: Epicatechin; EGC: Epigallocatechin; ECG: Epicatechin gallate; GCG: Gallocatechin gallate; DP: Dried plum; DA: Dried apple; AB: Alcoholic beer; NAB: Dealcoholized beer; DRW: Dealcoholized red wine; RW: Red wine; Ca: Calcium; $^{\delta}$: geometric mean [95% CI]; **numbers in bold** are the changes estimated as the mean difference: (A) after – (B) before (intra-group) or treated – control or placebo or comparator (between groups).

Table S5. Changes in LDL-cholesterol as reported in human randomized clinical trials (RCTs, parallel or crossover design) looking at the effects of (poly)phenol-containing products in postmenopausal women, without hormone replacement therapy or lipid-lowering therapy.

References	Health status	Treatment Groups (*N* participants per group)	(Poly)phenols	Dose (poly)phenols (mg /d)	Duration (d)	Total LDL-cholesterol (mg/dL)				
						Control/Placebo/ Comparator	Δ (A – B) (*p*-value)	Treatment	Δ (A – B) (*p*-value)	Δ (Treatment – Control/Placebo) (*p*-value)
**Cheng et al., 2004 [36]**	Healthy	Capsules<br><br>Estrogen group (*11*)<br>Isoflavone group (*17*)	Isoflavones (daidzein and genistein) in evening primrose oil	100	90<br>180	B: 130.9 ± 28.9<br>A90: 131.2 ± 28.9<br>A180: 149.4 ± 34.4	**+0.39**<br>**+18.5**<br>(NS)	B: 106.2 ± 30.1<br>A90: 124.7 ± 23.9<br>A180: 134.3 ± 26.6	**+18.5**<br>**+28.2**<br>(NS)	**+18.1**<br>**+9.6**<br>(NS)
**Sathyapalan et al., 2018 [37]**	Healthy	Snack bars (soy protein free isoflavones or with isoflavones)<br><br>Placebo (*60*)<br>Treated (*60*)	Mix isoflavones	66	180	B: 140.9 ± 34.7<br>A: 137.8 ± 28.9	-3.1<br>(NI)	B: 140.9 ± 27.0<br>A: 138.9 ± 23.2	-1.9<br>(NI)	+1.2<br>(NS)

**Myasoedova et al., 2016 [35]**	Healthy	Mixed herbs capsules (500 mg of mix grape seeds, green tea leaves, hop cone and garlic powder)<br><br>Placebo (*71*)<br>Treated ×3 capsules/d (*56*)	Mix (poly)phenols (procyanidin, genistein, daidzein, flavones, resveratrol, other phenolics)	283	365	B: 153.0 ± 42.0<br>A: NI	-8.0<br>(NS)	B: 170.0 ± 47.0<br>A: NI	-13.0<br>(0.040)	**-5.0**<br>(NS)
**Wu et al., 2012 [38]**	Healthy	Green tea extract capsules<br><br>Placebo (*32*)<br>T_1: Group G400 (*37*)<br>T_2: Group G800 (*34*)	Mix (poly)phenols (mostly EGCG plus EC, EGC, ECG, and GCG)	T_1: 400<br>T_2: 800	60	B: 127.0 [114.0, 142.0]$^{\delta}$<br>A: 128.0 [116.0, 141.0]$^{\delta}$	**+1.0**<br>(NS)	**T_1: Group G400**<br>B: 129.0 [117.0, 143.0]$^{\delta}$<br>A: 119.0 [109.0, 130.0]$^{\delta}$<br><br>**T_2: Group G800**<br>B: 122.0 [110.0, 136.0]$^{\delta}$<br>A: 114.0 [104.0, 126.0]$^{\delta}$	**G400**<br>**-10.0**<br>(0.007)<br><br>**G800**<br>**-8.0**<br>(0.012)	**Group G400 *vs* P**<br>**-11.0**<br>(0.021)<br><br>**Group G800 *vs* P**<br>**-9.0**<br>(0.021)<br><br>**Groups *vs* P**<br>NI<br>(NS)<br><br>**Group G800 *vs* G400**<br>**+2.0**<br>(NS)
**Curtis et al., 2009 [39]**	Healthy	Elderberry extract capsules (125 mg)<br><br>Placebo (*26*)<br>Treated (*26*)	Mix anthocyanins (mostly cyanidin-3-glucoside) per capsule	500	84	B: 135.1 ± 23.2<br>A: 127.4 ± 30.9	**-7.7**<br>(NS)	B: 131.2 ± 23.2<br>A: 131.2 ± 27.0	**0.0**<br>(NS)	**+7.7**<br>(NS)
**Zern et al., 2005 [40]**	Healthy	Lyophilized grape powder<br><br>Placebo (*20*)<br>Treated (*20*)	Mix grape (poly)phenols (anthocyanins, quercetin, myricetin, kaempferol and resveratrol)	~210	28	B: 111.9 ± 33.6<br>A: 108.1 ± 31.7	**-3.9**<br>(NS)	B: 111.9 ± 33.6<br>A: 104.2 ± 35.5	**-7.7**<br>(NS)	**-3.9**<br>(<0.050)
**Chai et al., 2012 [41]**	Healthy	Dried plum or dried apple<br><br>T_1: Group DP (*55*)<br>T_2: Group DA (*45*)	Mix (poly)phenols	NI	90<br>180<br>365	**T_1: Group DP**<br>B: 100.4 ± 87.0<br>A90: 96.5 ± 69.0<br>A180: 97.3 ± 78.0<br>A365: 93.4 ± 78.0	 **-3.9**<br>**-3.1**<br>**-6.9**<br>(NS)	**T_2: Group DA**<br>B: 110.4 ± 70.0<br>A90: 93.4 ± 55.0<br>A180: 83.4 ± 55.0<br>A365: 85.3 ± 78.0	 **-16.9**<br>**-27.0**<br>**-25.1**<br>(0.002)	**Group DA *vs* DP**<br>**-13.1**<br>**-23.9**<br>**-18.1**<br>(NS)

**Al-Dashi et al., 2019 [34]**	Healthy	Prunes<br><br>T_1: Low dose 14 g (*27*)<br>T_2: High dose 42 g (*27*)	Mix (poly)phenols (chlorogenic, neochlorogenic acids, others)	NI	14	B: 127.0^{δ} ± 37.0<br>A: 126.0^{δ} ± 34.0	-0.60<br>(NS)	B: 128.0^{δ} ± 37.0<br>A: 128.0^{δ} ± 31.0	-0.10<br>(NS)	+0.50<br>(NS)
**García-Yu et al., 2020 [42]**	Healthy	10g of dark chocolate (99% cocoa)<br><br>Control (*66*)<br>Treated (*71*)	Flavanols	65	180	B: 122.0 ± 26.9<br>A: 124.0 ± 29.3	+1.6<br>(NS)	B: 128.0 ± 26.4<br>A: 130.0 ± 29.1	+2.7<br>(NS)	+1.5<br>(NS)
**Estévez-Santiago et al., 2019 [44]**	Healthy	Capsules<br><br>T_1: Group X *(26)*<br>T_2: Group A *(23)*<br>T_3: Group A+X *(23)*	T_1: Xanthophylls (lutein+zeaxanthin)<br>T_2: Anthocyanins<br>T_3: Anthocyanins and xanthophylls (lutein+zeaxanthin)	T_1: 8.0<br>T_2: 60<br>T_3: 68	120<br>240	**T_1: Group X**<br>B: 143.9 ± 29.7<br>A120: NI<br>A240: NI	 NI<br>NI<br>(NS)	**T_2: Group A**<br>B: 145.5 ± 42.5<br>A120: NI<br>A240: NI<br><br>**T_3: Group A+X**<br>B: 150.9 ± 23.5<br>A120: NI<br>A240: NI	 NI<br>NI<br>(NS)<br><br><br>NI<br>NI<br>(NS)	**Group A *vs*** X<br>NI<br>NI<br>(NS)<br><br>**Group A+X *vs* X**<br>NI<br>NI<br>(NS)<br><br>**Group A+X *vs* A**<br>NI<br>NI<br>(NS)
**Trius-Soler et al., 2021 [32]**	Healthy	Beer beverage with and without alcohol<br>Control (*14*)<br>T_1: Group AB (*16*)<br>T_2: Group NAB (*7*)	Prenylflavonoids	T_1: 0.359<br>T_2: 0.259	180	 B: 114.0 ± 23.0<br>A: NI	 NI<br>(NS)	**T_1: Group AB**<br>B: 135.0 ± 25.0<br>A:NI<br><br><br>**T_2: Group NAB**<br>B: 142.0 ± 18.0<br>A: NI	 NI<br>(NS)<br><br><br><br>NI<br>(NS)	**Group AB *vs* C**<br>-12.8<br>(NS)<br><br><br>**Group NAB *vs* C**<br>-16.1<br>(0.016)<br><br>**Groups *vs* C**<br>NI<br>(NS)<br><br>**Group NAB *vs* AB**<br>-3.3<br>(NS)

**Wang-Polagruto et al., 2006 [25]**	Dyslipidaemia (high cholesterol)	Flavanol cocoa beverage<br><br>T_1: Low flavanol (*16*)<br>T_2: High flavanol (*16*)	Mix flavanols	T_1: 43<br>T_2: 446	42	**T_1: Low flavanol**<br>B: 150.0 ± 36.0<br>A: NI	 NI<br>(NS)	**T_2: High flavanol**<br>B: 149.0 ± 20.0<br>A: NI	 NI<br>(NS)	**High *vs* Low**<br>NI<br>(NS)
**Naissides et al., 2006b [27]**	Dyslipidaemia (high cholesterol)	Red wine RW (2.5 g (poly)phenols/L)<br><br>Control (400 mL water) (*16*)<br>T_1: Group DRW non-alcohol (400 mL) (*15*)<br>T_2: Group RW alcohol (400 mL) (*14*)	Mix (poly)phenols	1000	42	 NI<br>(NS)	 NI<br>(NS)	**T_1: Group DRW**<br>B: NI<br>A: NI<br><br>**T_2: Group RW**<br>B: NI<br>A: NI	**DRW**<br>+0.13‡<br>(NS)<br><br>**RW**<br>-8.0‡<br>(NS)	**Group DRW *vs* C**<br>**-0.40**<br>(NS)<br><br>**Group RW *vs* C**<br>**-9.0**<br>(<0.050)<br><br>**Groups *vs* C**<br>NI<br>(NS)<br><br>**Group DRW *vs* RW**<br>**-8.0**<br>(NS)
**Aubertin-Leheudre et al., 2008 [45]**	Obese	Soybeans capsules (17.5 mg)<br><br>Placebo (*18*)<br>Treated (*21*)	Mix isoflavones (daidzein, glycitein, and genistein)	70	180	B: 122.4 ± 30.1<br>A: 134.3 ± 25.1	**-11.9**<br>(NS)	B: 122.4 ± 31.3<br>A: 122.4 ± 26.6	**0.0**<br>(NS)	**+11.9**<br>(NS)
**Filip et al., 2015 [28]**	Osteopenia (mix cholesterol levels)	Olive leaf extract<br><br>Placebo (400 mg Ca capsules) (*21*)<br>Treated (400 mg Ca + 250 mg olive extract capsules) (*27*)	Mix (poly)phenols (>40% oleuropein)	>100 oleuropein	180<br>365	B: 174.4 ± 37.6<br>A180: 143.4 ± 30.0<br>A365: 154.1 ± 30.5	**-30.9**<br>-20.4<br>(0.020)	B: 167.2 ± 51.2<br>A180: 145.5 ± 38.6<br>A365: 132.5 ± 29.7	**-21.7**<br>-34.7<br>(0.020)	**+9.3**<br>**+14.3**<br>(NS)

Data are presented as the mean ± SD unless otherwise stated; B: Before intervention; A: After intervention; NS: Not significant: NI: Not indicated; C: Control; T: Treated; P: Placebo; EGCG: Epigallocatechin gallate; EC: Epicatechin; EGC: Epigallocatechin; ECG: Epicatechin gallate; GCG: Gallocatechin gallate; DP: Dried plum; DA: Dried apple; AB: Alcoholic beer; NAB: Dealcoholized beer; DRW: Dealcoholized red wine; RW: Red wine; Ca: Calcium; ‡: % change reported by the authors; $^{\delta}$: geometric mean [95% CI]; **numbers in bold** are the changes estimated as the mean difference: (A) after – (B) before (intra-group) or treated – control or placebo or comparator (between groups).

Table S6. Changes in HDL as reported in human RCTs (parallel or crossover design) looking at the chronic effects of (poly)phenol-containing products in postmenopausal women, without hormone replacement therapy or lipid-lowering therapy.

References	Health status	Treatment Groups (*N* participants per group)	(Poly)phenols	Dose (poly)phenols (mg /d)	Duration (d)	Total circulating HDL (mg/dL)				
						Control/Placebo/ Comparator	Δ (A – B) (*p*-value)	Treatment	Δ (A – B) (*p*-value)	Δ (Treatment – Control/Placebo) (*p*-value)
**Cheng et al., 2004 [36]**	Healthy	Capsules<br><br>Estrogen (*11*)<br>Isoflavone (*17*)	Isoflavones (daidzein and genistein) in evening primrose oil	100	90<br>180	B: 60.2 ± 14.3<br>A90: 66.0 ± 18.1<br>A180: 65.2 ± 16.9	**+5.8**<br>**+5.0**<br>(NS)	B: 71.0 ± 29.3<br>A90: 64.8 ± 22.8<br>A180: 65.6 ± 20.1	**-6.2**<br>**-5.4**<br>(NS)	**-11.9**<br>**-10.4**<br>(NS)
**Sathyapalan et al., 2018 [37]**	Healthy	Snack bars (soy protein free isoflavones or with isoflavones)<br><br>Placebo (*60*)<br>Treated (*60*)	Mix isoflavones	66	180	B: 68.7 ± 16.2<br>A: 63.7 ± 15.1	**-5.0**<br>(NI)	B: 64.8 ± 36.3<br>A: 62.5 ± 13.9	**-2.3**<br>(NI)	**+2.7**<br>(NS)
**Myasoedova et al., 2016 [35]**	Healthy	Mixed herbs capsules (500 mg of mix grape seeds, green tea leaves, hop cone and garlic powder)<br><br>Placebo (*71*)<br>Treated ×3 capsules/d (*56*)	Mix (poly)phenols (procyanidin, genistein, daidzein, flavones, resveratrol, other phenolics)	283	365	B: 74.0 ± 18.0<br>A: NI	-3.0<br>(0.038)	B: 74.0 ± 15.0<br>A: NI	-3.0<br>(NS)	NI<br>(NS)
**Wu et al., 2012 [38]**	Healthy	Green tea extract capsules<br>Placebo (*32*)<br>T_1: Group G400 (*37*)<br>T_2: Group G800 (*34*)	Mix (poly)phenols (mostly EGCG plus EC, EGC, ECG, and GCG)	T_1: 400<br>T_2: 800	60	B: 61.0 [56.0, 67.0]$^{\delta}$<br>A: 61.0 [56.0, 67.0]$^{\delta}$	**0.0**<br>(NS)	**T_1: Group G400**<br>B: 62.0 [57.0, 67.0]$^{\delta}$<br>A: 62.0 [57.0, 67.0]$^{\delta}$<br><br>**T_2: Group G800**<br>B: 59.0 [54.0, 63.0]$^{\delta}$<br>A: 58.0 [53.0, 63.0]$^{\delta}$	**G400**<br>**0.0**<br>(NS)<br><br>**G800**<br>**-10.0**<br>(NS)	**Group G400 *vs* P**<br>**0.0**<br>(NS)<br><br>**Group G800 *vs* P**<br>**-10.0**<br>(NS)<br><br>**Groups *vs* P**<br>NI<br>(NS)<br><br>**Group G800 *vs* G400**<br>**-10.0**<br>(NS)

**Curtis et al., 2009 [39]**	Healthy	Elderberry extract capsules (125 mg)<br>Placebo (*26*)<br>Treated (*26*)	Mix anthocyanins (mostly cyanidin-3-glucoside) per capsule	500	84	B: 61.8 ± 11.6<br>A: 61.8 ± 11.6	**0.0**<br>(NS)	B: 61.8 ± 15.4<br>A: 61.8 ± 15.4	**0.0**<br>(NS)	**0.0**<br>(NS)
**Zern et al., 2005 [40]**	Healthy	Lyophilized grape powder<br><br>Placebo (*20*)<br>Treated (*20*)	Mix grape (poly)phenols (anthocyanins, quercetin, myricetin, kaempferol and resveratrol)	~210	28	B: 69.5 ± 13.5<br>A: 73.3 ± 14.3	**+3.9**<br>(NS)	B: 69.5 ± 13.5<br>A: 73.3 ± 13.1	**+3.9**<br>(NS)	**0.0**<br>(NS)
**Chai et al., 2012 [41]**	Healthy	Dried plum or dried apple<br><br>T_1: Group DP (*55*)<br>T_2: Group DA (*45*)	Mix (poly)phenols	NI	90<br>180<br>365	**T_1: Group DP**<br>B: 67.0 ± 16.0<br>A90: 68.0 ± 15.0<br>A180: 68.0 ± 15.0<br>A365: 67.0 ± 15.0	 **+1.0**<br>**+1.0**<br>**0.0**<br>(NS)	**T_2: Group DA**<br>B: 62.0 ± 15.0<br>A90: 64.0 ± 15.0<br>A180: 62.0 ± 15.0<br>A365: 64.0 ± 19.0	 **+2.0**<br>**0.0**<br>**+2.0**<br>(NS)	**Group DA *vs* DP**<br>**+1.0**<br>**-1.0**<br>**+2.0**<br>(NS)
**Al-Dashti et al., 2019 [34]**	Healthy	Prunes<br><br>T_1: Low dose 14 g (*27*)<br>T_2: High dose 42 g (*27*)	Mix (poly)phenols (chlorogenic, neochlorogenic acids, others)	NI	14	**T_1: Low dose**<br>B: 63.0^{δ} ± 23.0<br>A: 63.0^{δ} ± 23.0	 **0.0**<br>(NS)	**T_2: High dose**<br>B: 62.0^{δ} ± 23.0<br>A: 62.0^{δ} ± 22.0	 **-0.90**<br>(NS)	**High *vs* Low**<br>**-0.90**<br>(NS)
**Garcia-Yu et al., 2020 [42]**	Healthy	10g of dark chocolate (99% cocoa)<br><br>Control (*66*)<br>Treated (*71*)	Flavanols	65	180	B: 65.8 ± 13.2<br>A: 65.0 ± 12.9	-0.90<br>(NS)	B: 68.2 ± 17.3<br>A: 67.0 ± 15.9	-1.2<br>(NS)	-0.32<br>(NS)
**Estévez-Santiago et al., 2019 [44]**	Healthy	Capsules<br><br>T_1: Group X (*26*)<br>T_2: Group A (*23*)<br>T_3: Group A+X (*23*)	T_1: Xanthophylls (lutein+zeaxanthin)<br>T_2: Anthocyanins<br>T_3: Anthocyanins and xanthophylls (lutein+zeaxanthin)	T_1: 8.0<br>T_2: 60<br>T_3: 68	120<br>240	**T_1: Group X**<br>B: 66.4 ± 12.7<br>A120: NI<br>A240: NI	 NI<br>NI<br>(NS)	**T_2: Group A**<br>B: 63.3 ± 12.7<br>A120: NI<br>A240: NI<br><br>**T_3: Group A+X**<br>B: 71.4 ± 13.5<br>A120: NI<br>A240: NI	 NI<br>NI<br>(NS)<br><br><br>NI<br>NI<br>(NS)	**Group A *vs* X**<br>NI<br>NI<br>(NS)<br><br>**Group A+X *vs* X**<br>NI<br>NI<br>(NS)<br><br>**Group A+X *vs* A**<br>NI<br>NI<br>(NS)

**Trius-Soler et al., 2021 [44]**	Healthy	Beer beverage with and without alcohol<br>Control (*14*)<br>T_1: Group AB (*16*)<br>T_2: Group NAB (*7*)	Prenylflavonoids	T_1: 0.359<br>T_2: 0.259	180	B: 56.0 ± 13.0<br>A: NI	NI<br>(NS)	**T_1: Group AB**<br>B: 57.0 ± 8.0<br>A:NI<br><br>**T_2: Group NAB**<br>B: 56.0 ± 13.0<br>A: NI	NI<br>(NS)<br><br><br>NI<br>(NS)	**Group AB *vs* C**<br>+3.5<br>(NS)<br><br>**Group NAB *vs* C**<br>+1.3<br>(NS)<br><br>**Groups *vs* C**<br>NI<br>(NS)<br><br>**Group NAB *vs* AB**<br>-2.2<br>(NS)
**Wang-Polagruto et al., 2006 [25]**	Dyslipidaemia (high cholesterol)	Flavanol cocoa beverage<br>T_1: Low flavanol (*16*)<br>T_2: High flavanol (*16*)	Mix flavanols	T_1: 43<br>T_2: 446	42	B: 68.1 ± 45.0<br>A: 61.7 ± NI	**-6.5**<br>(<0.050)	B: 69.7 ± 19.0<br>A: 74.3 ± NI	**+4.6**<br>(<0.050)	**+11.1**<br>(<0.050)
**Filip et al., 2015 [28]**	Osteopenia (mix cholesterol levels)	Olive leaf extract<br>Placebo (400 mg Ca capsules) (*21*)<br>Treated (400 mg Ca + 250 mg olive extract capsules) (*27*)	Mix (poly)phenols (>40% oleuropein)	>100 oleuropein	180<br>365	B: 50.1 ± 9.7<br>A180: 51.1 ± 6.0<br>A365: 51.4 ± 9.3	**+1.0**<br>+1.3<br>(365d)<br>(NS)	B: 48.2 ± 10.2<br>A180: 51.6 ± 8.1<br>A365: 53.9 ± 8.9	**+3.4**<br>+5.6<br>(365d)<br>(NS)	**+2.3**<br>**+4.3**<br>(NS)
**D´Anna et al., 2014 [31]**	Diagnosis of metabolic syndrome	Capsules (30 mg of cocoa (poly)phenols, 80 mg of soy isoflavones, and 2g of myo-inositol)<br><br>Placebo (*21*)<br>Treated (*22*)	Cocoa (poly)phenols and of soy isoflavones	110	180	B: 45.0 ± 6.0<br>A: 46.0 ± 8.0	**+1.0**<br>(NS)	B: 44.0 ± 7.0<br>A: 50.0 ± 6.0	**+6.0**<br>(NS)	**+5.0**<br>(NS)
**Naissides et al., 2006b [27]**	Dyslipidaemia (high cholesterol)	Red wine RW (2.5 g (poly)phenols/L)<br><br>Control (400 mL water) (*16*)<br><br>T_1: Group DRW non-alcohol (400 mL) (*15*)	Mix (poly)phenols	1000	42	B: NI<br>A: NI	+7.1‡<br>(NI)	**T_1: Group DRW**<br>B: NI<br>A: NI<br><br>**T_2: Group RW**<br>B: NI<br>A: NI	**DRW**<br>-4.7‡<br>(NS)<br><br>**RW**<br>+17.6‡<br>(<0.050)	**Group DRW *vs* C**<br>NI<br>(NS)<br><br>**Group RW *vs* C**<br>NI<br>(NS)

		T_2: Group RW alcohol (400 mL) (*14*)								**Groups** ***vs*** **C**<br>NI<br>(NI)<br><br>**Group DRW** ***vs*** **RW**<br>NI<br>(NS)
**Aubertin-Leheudre et al., 2008 [45]**	Obese	Soybeans capsules (17.5 mg)<br>Placebo (*18*)<br>Treated (*21*)	Mix isoflavones (daidzein, glycitein, and genistein)	70	180	B: 55.9 ± 14.3<br>A: 59.8 ± 13.1	**+3.9**<br>(NS)	B: 59.8 ± 18.9<br>A: 54.4 ± 11.2	**-5.4**<br>(NS)	**-9.3**<br>(NS)

Data are presented as the mean ± SD unless otherwise stated; B: Before intervention; A: After intervention; NS: Not significant: NI: Not indicated; C: Control; T: Treated; P: Placebo; EGCG: Epigallocatechin gallate; EC: Epicatechin; EGC: Epigallocatechin; ECG: Epicatechin gallate; GCG: Gallocatechin gallate; DP: Dried plum; DA: Dried apple; AB: Alcoholic beer; NAB: Dealcoholized beer; DRW: Dealcoholized red wine; RW: Red wine; Ca: Calcium; ‡: % change reported by the authors; $^{\delta}$: geometric mean [95% CI]; **numbers in bold** are the changes estimated as the mean difference: (A) after – (B) before (intra-group) or treated – control or placebo or comparator (between groups).

Table S7. Changes in total triglycerides as reported in human RCTs (parallel or crossover design) looking at the chronic effects of (poly)phenol-containing products in postmenopausal women, without hormone replacement therapy or lipid-lowering therapy.

**References**	**Health status**	**Treatment Groups (*N* participants per group)**	**(Poly)phenols**	**Dose (poly)phenols (mg /d)**	**Duration (d)**	**Total circulating triglycerides (mg/dL)**				
						**Control/Placebo/ Comparator**	**Δ (A – B) (*p*-value)**	**Treatment**	**Δ (A – B) (*p*-value)**	**Δ (Treatment – Control/Placebo) (*p*-value)**
**Cheng et al., 2004 [36]**	Healthy	Capsules<br><br>Estrogen (*11*)<br>Isoflavone (*17*)	Isoflavones (daidzein and genistein) in evening primrose oil	100	90<br>180	B: 122.2 ± 52.3<br>A90: 147.1 ± 74.4<br>A180: 139.1 ± 107.2	**+24.8**<br>**+16.9**<br>(NS)	B: 111.6 ± 43.4<br>A90: 127.6 ± 73.5<br>A180: 113.4 ± 48.7	**+15.9**<br>**+1.8**<br>(NS)	**-8.9**<br>**-15.1**<br>(NS)
**Sathyapalan et al., 2018 [37]**	Healthy	Snack bars (soy protein free isoflavones or with isoflavones)<br><br>Placebo (*60*)<br>Treated (*60*)	Mix isoflavones	66	180	B: 104.5 ± 50.5<br>A: 112.5 ± 80.6	**+7.9**<br>(NS)	B: 102.8 ± 47.8<br>A: 108.1 ± 62.9	**+5.3**<br>(NS)	**-2.7**<br>(NS)
**Myasoedova et al., 2016 [35]**	Healthy	Mixed herbs capsules (500 mg of mix grape seeds, green tea leaves, hop cone and garlic powder)<br><br>Placebo (*71*)<br>Treated ×3 capsules/d (*56*)	Mix (poly)phenols (procyanidin, genistein, daidzein, flavones, resveratrol, other phenolics)	283	365	B: 126.0 ± 51.0<br>A: NI	-9.0<br>(NS)	B: 134.0 ± 78.0<br>A: NI	-9.0<br>(NS)	NI<br>(NS)
**D'Anna et al., 2014 [31]**	Diagnosis of metabolic syndrome	Capsules (30 mg of cocoa (poly)phenols, 80 mg of soy isoflavones, and 2g of myo-inositol)<br><br>Placebo (*21*)<br>Treated (*22*)	Cocoa (poly)phenols and of soy isoflavones	110	180	B: 180.0 ± 20.0<br>A: 165.0 ± 18.0	**-15.0**<br>(0.008)	B: 177.0 ± 19.0<br>A: 145.0 ± 14.0	**-32.0**<br>(<0.001)	**-17.0**<br>(<0.001)
**Wu et al., 2012 [38]**	Healthy	Green tea extract capsules<br><br>Placebo (*32*)<br>T_1: Group G400 (*37*)<br>T_2: Group G800 (*34*)	Mix (poly)phenols (mostly EGCG plus EC, EGC, ECG, and GCG)	T_1: 400<br>T_2: 800	60	 B: 106.0 [91.0, 124.0]$^{\delta}$<br>A: 109.0 [94.0, 127.0]$^{\delta}$	 **+3.0**<br>(NS)	**T_1: Group G400**<br>B: 107.0 [93.0, 123.0]$^{\delta}$<br>A: 106.0 [93.0, 121.0]$^{\delta}$<br><br>**T_2: Group G800**<br>B: 108.0 [93.0, 125.0]$^{\delta}$	**G400**<br>**-1.0**<br>(NS)<br><br>**G800**<br>**+10.0**	**Group G400 *vs* P**<br>**-4.0**<br>(NS)<br><br>**Group G800 *vs* P**<br>**+7.0**

								A: 118.0 [103.0, 136.0]$^{\delta}$	(0.042)	(NS)<br>**Groups *vs* P**<br>NI<br>(NS)<br><br>**Group G800 *vs* G400**<br>**+13**<br>NI<br>(0.090)
**Curtis et al., 2009 [39]**	Healthy	Elderberry extract capsules (125 mg)<br><br>Placebo (*26*)<br>Treated (*26*)	Mix anthocyanins (mostly cyanidin-3-glucoside) per capsule	500	84	B: 79.7 ± 26.6<br>A: 88.6 ± 35.4	**+8.9**<br>(NS)	B: 79.7 ± 26.6<br>A: 88.6 ± 26.6	**+8.9**<br>(NS)	**0.0**<br>(NS)
**Zern et al., 2005 [40]**	Healthy	Lyophilized grape powder<br><br>Placebo (*20*)<br>Treated (*20*)	Mix grape (poly)phenols (anthocyanins, quercetin, myricetin, kaempferol and resveratrol)	~210	28	B: 132.9 ± 106.3<br>A: 150.6 ± 88.6	**+17.7**<br>(NS)	B: 132.9 ± 106.3<br>A: 141.8 ± 97.45	**+8.9**<br>(NS)	**-8.9**<br>(0.002)
**Chai et al., 2012 [41]**	Healthy	Dried plum or dried apple<br><br>T1: Group DP (*55*)<br>T2: Group DA (*45*)	Mix (poly)phenols	NI	90<br>180<br>365	**T1: Group DP**<br>B: 103.0 ± 13.0<br>A90: 106.0 ± 33.0<br>A180: 111.0 ± 38.0<br>A365: 111.0 ± 34.0	 **+3.0**<br>**+8.0**<br>**+8.0**<br>(NS)	**T2: Group DA**<br>B: 115.0 ± 38.9<br>A90: 110.0 ± 34.8<br>A180: 118.0 ± 45.5<br>A365: 104.0 ± 33.5	 **-5.0**<br>**-9.0**<br>**-9.0**<br>(NS)	**Group DA *vs* DP**<br>**-8.0**<br>**-17.0**<br>**-17.0**<br>(NS)
**Al-Dashti et al., 2019 [34]**	Healthy	Prunes<br><br>T1: Low dose 14 g (*27*)<br>T2: High dose 42 g (*27*)	Mix (poly)phenols (chlorogenic, neochlorogenic acids, others)	NI	14	**T1: Low dose**<br>B: 99.0$^{\delta}$ ± 107.0<br>A: 93.0$^{\delta}$ ± 96.0	 -6.0<br>(NS)	**T2: High dose**<br>B: 91.0$^{\delta}$ ± 71.0<br>A: 95.0$^{\delta}$ ± 91.0	 +5.0<br>(NS)	**High *vs* Low**<br>+10.0<br>(NS)
**García-Yu et al., 2020 [42]**	Healthy	10g of dark chocolate (99% cocoa)<br><br>Control (*66*)<br>Treated (*71*)	Flavanols	65	180	B: 80.0 ± 34.3<br>A: 80.3 ± 28.5	+0.20<br>(NS)	B: 83.4 ± 30.6<br>A: 83.1 ± 34.7	-0.30<br>(NS)	-0.49<br>(NS)

**Estévez-Santiago et al., 2019 [44]**	Healthy	Capsules<br><br>T_1: Group X *(26)*<br>T_2: Group A *(23)*<br>T_3: Group A+X *(23)*	T_1: Xanthophylls (lutein+zeaxanthin)<br>T_2: Anthocyanins<br>T_3: Anthocyanins and xanthophylls (lutein + zeaxanthin)	T_1: 8.0<br>T_2: 60<br>T_3: 68	120<br>240	**T_1: Group X**<br>B: 84.2 ± 33.7<br>A120: NI<br>A240: NI	 NI<br>NI<br>(NS)	**T_2: Group A**<br>B: 92.1 ± 32.8<br>A120: NI<br>A240: NI<br><br>**T_3: Group A+X**<br>B: 81.5 ± 26.6<br>A120: NI<br>A240: NI	 NI<br>NI<br>(NS)<br><br><br>NI<br>NI<br>(NS)	**Group A *vs X***<br>NI<br>NI<br>(NS)<br><br>**Group A+X *vs* X**<br>NI<br>NI<br>(NS)<br><br>**Group A+X *vs* A**<br>NI<br>NI<br>(NS)
**Trius-Soler et al., 2021 [32]**	Healthy	Beer beverage with and without alcohol<br><br>Control *(14)*<br>T_1: Group AB *(16)*<br>T_2: Group NAB *(7)*	Prenylflavonoids	T_1: 0.359<br>T_2: 0.259	180	B: 69.0 ± 32.0<br>A: NI	 NI<br>(NS)	**T_1: Group AB**<br>B: 77.0 ± 25.0<br>A: NI<br><br>**T_2: Group NAB**<br>B: 66.0 ± 18.0<br>A: NI	 NI<br>(NS)<br><br><br>NI<br>(NS)	**Group AB *vs* C**<br>+7.2<br>(NS)<br><br>**Group NAB *vs* C**<br>+5.3<br>(NS)<br><br>**Groups *vs* C**<br>NI<br>(NS)<br><br>**Group NAB *vs* AB**<br>-1.9<br>(NS)
**Wang-Polagruto et al., 2006 [25]**	Dyslipidaemia (high cholesterol)	Flavanol cocoa beverage<br><br>T_1: Low flavanol *(16)*<br>T_2: High flavanol *(16)*	Mix flavanols	T_1: 43<br>T_2: 446	42	**T_1: Low flavanol**<br>B: 111.1 ± 85.0<br>A: NI	 NI<br>(NS)	**T_2: High flavanol**<br>B: 79.7 ± 31.0<br>A: NI	 NI<br>(NS)	**High *vs* Low**<br>NI<br>(NS)
**Naissides et al., 2006b [27]**	Dyslipidaemia (high cholesterol)	Red wine RW (2.5 g (poly)phenols/L)<br><br>Control (400 mL water) *(16)*	Mix (poly)phenols	1000	42	 B: 109.9 ± 43.0<br>A: 107.2 ± 35.0	 **-2.7**<br>(NS)	**T_1: Group DRW**<br>B: 117.8 ± 59.0<br>A: 116.1 ± 48.0<br><br>**T_2: Group RW**	**DRW**<br>**-1.8**<br>(NS)<br><br>**RW**	**Group DRW *vs* C**<br>**-0.89**<br>(NS)<br><br>**Group RW *vs* C**

		T_1: Group DRW non-alcohol (400 mL) (*15*)<br>T_2: Group RW alcohol (400 mL) (*14*)						B: 105.4 ± 50.0<br>A: 114.3 ± 59.0	**+8.9**<br>(NS)	**+11.5**<br>(NS)<br><br>**Groups *vs* C**<br>NI<br>(NS)<br><br>**Group DRW *vs* RW**<br>**- 10.6**<br>(NS)
**Aubertin-Leheudre et al., 2008 [45]**	Obese	Soybeans capsules (17.5 mg)<br><br>Placebo (*18*)<br>Treated (*21*)	Mix isoflavones (daidzein, glycitein, and genistein)	70	180	B: 134.7 ± 61.0<br>A: 118.7 ± 52.0	**-15.9**<br>(NS)	B: 133.8 ± 61.0<br>A: 125.8 ± 52.0	**-7.9**<br>(NS)	**+7.9**<br>(NS)
**Filip et al., 2015 [28]**	Osteopenia (mix cholesterol levels)	Olive leaf extract<br><br>Placebo (400 mg Ca capsules) (*21*)<br>Treated (400 mg Ca + 250 mg olive extract capsules) (*27*)	Mix (poly)phenols (>40% oleuropein)	>100 oleuropein	180<br>365	B: 122.2 ± 59.6<br>A180: 130.9 ± 60.1<br>A365: 141.6 ± 77.4	**+8.7**<br>+18.9<br>(365d)<br>(0.010)	B: 98.8 ± 42.9<br>A180: 104.6 ± 59.9<br>A365: 94.7 ± 37.5	**+5.8**<br>-4.2<br>(365d)<br>(0.010)	**-2.9**<br>-23.0<br>(NS)

Data are presented as the mean ± SD unless otherwise stated; B: Before intervention; A: After intervention; NS: Not significant: NI: Not indicated; C: Control; T: Treated; P: Placebo; EGCG: Epigallocatechin gallate; EC: Epicatechin; EGC: Epigallocatechin; ECG: Epicatechin gallate; GCG: Gallocatechin gallate; DP: Dried plum; DA: Dried apple; AB: Alcoholic beer; NAB: Dealcoholized beer; DRW: Dealcoholized red wine; RW: Red wine; Ca: Calcium; $^{\delta}$: geometric mean [95% CI]; **numbers in bold** are the changes estimated as the mean difference: (A) after – (B) before (intra-group) or treated – control or placebo or comparator (between groups).

Table S8. Changes in systolic and diastolic blood pressure as reported in human RCTs (parallel or crossover design) looking at the chronic effects of (poly)phenol-containing products in postmenopausal women, without hormone replacement therapy.

References	Health status	Treatment Groups (*N* participants per group)	(Poly)phenols	Dose (poly)phenols (mg /d)	Duration (d)	Blood pressure (mmHg)				
						Control/Placebo/ Comparator	Δ (A – B) (*p*-value)	Treatment	Δ (A – B) (*p*-value)	Δ (Treatment – Control/Placebo) (*p*-value)
**Sathyapalan et al., 2018 [37]**	Healthy	Snack bars (soy protein free isoflavones or with isoflavones)	Mix isoflavones	66	180	***Systolic***				
						B: 124.6 ± 18.8<br>A: 123.4 ± 16.0	-0.80<br>(NI)	B: 125.0 ± 20.2<br>A: 121.2 ± 14.9	-3.2<br>(NI)	-2.5<br>(<0.01)
						Diastolic				
		Placebo (*60*)<br>Treated (*60*)				B: 77.2 ± 10.9<br>A: 77.4 ± 11.6	+0.20<br>(NI)	B: 77.0 ± 13.8<br>A: 76.8 ± 9.4	-0.60<br>(NI)	-0.80<br>(NS)
**Myasoedova et al., 2016 [35]**	Healthy	Mixed herbs capsules (500 mg of mix grape seeds, green tea leaves, hop cone and garlic powder)	Mix (poly)phenols (procyanidin, genistein, daidzein, flavones, resveratrol, other phenolics)	283	365	***Systolic***				
						B: 135.0 ± 18.0<br>A: NI	-1.0<br>(NS)	B: 127.0 ± 13.0<br>A: NI	+5.0<br>(0.051)	**+4.0**<br>(NS)
						Diastolic				
		Placebo (*71*)<br>Treated ×3 capsules/d (*56*)				B: 83.0 ± 9.0<br>A: NI	-1.0<br>(NS)	B. 79.0 ± 8.0<br>A: NI	-1.0<br>(NS)	**0.0**<br>(NS)
**Curtis et al., 2009 [39]**	Healthy	Elderberry extract capsules (125 mg)	Mix anthocyanins (mostly cyanidin-3-glucoside) per capsule	500	84	***Systolic***				
						B: 130.0 ± 14.0<br>A: 124.0 ± 15.0	**-6.0**<br>(NI)	B: 123.0 ± 15.0<br>A: 124.0 ± 13.0	**+1.0**<br>(NI)	**+7.0**<br>(NI)
						Diastolic				
		Placebo (*26*)<br>Treated (*26*)				B: 82.0 ± 11.0<br>A: 80.0 ± 10.0	**-2.0**<br>(NI)	B: 78.0 ± 7.0<br>A: 77.0 ± 7.0	**-1.0**<br>(NI)	**+1.0**<br>(NI)
**Al-Dashti et al., 2019 [34]**	Healthy	Prunes	Mix (poly)phenols (chlorogenic, neochlorogenic acids, others)	NI	14	***Systolic***				
		T_1: Low dose 14 g (*27*)<br>T_2: High dose 42 g (*27*)				**T_1: Low dose**		**T_2: High dose**		**High *vs* Low**
						B: 115.0$^{\delta}$ ± 21.0<br>A: 114.0$^{\delta}$ ± 18.0	-1.0<br>(NS)	B: 115.0$^{\delta}$ ± 18.0<br>A: 114.0$^{\delta}$ ± 18.0	**-0.80**<br>(NS)	**+0.20**<br>(NS)
						Diastolic				
						**T_1: Low dose**		**T_2: High dose**		**High *vs* Low**
						B: 75.0$^{\delta}$ ± 11.0<br>A: 75.0$^{\delta}$ ± 11.0	**0.0**<br>(NS)	B: 76.0$^{\delta}$ ± 10.0<br>A: 74.0$^{\delta}$ ± 10.0	**-2.0**<br>(NS)	**-2.0**<br>(NS)
**Garcia-Yu et al., 2020 [42]**	Healthy	10 g of dark chocolate (99% cocoa)	Flavanols	65	180	***Systolic***				
		Control (*66*)				B: 108.0 ± 15.0<br>A: 108.0 ± 14.4	-0.20<br>(NS)	B: 108.0 ± 16.4<br>A: 106.0 ± 14.1	-1.8<br>(NS)	-1.45<br>(NS)

		Treated (*71*)								
						Diastolic				
						B: 72.2 ± 10.3<br>A: 71.4 ± 10.1	-0.70<br>(NS)	B: 72.6 ± 10.7<br>A: 72.4 ± 10.0	-0.30<br>(NS)	+0.59<br>(NS)
**Estévez-Santiago et al., 2019 [44]**	Healthy	Capsules<br><br>T_1: Group X (*26*)<br>T_2: Group A (*23*)<br>T_3: Group A+X (*23*)	T_1: Xanthophylls (lutein + zeaxanthin)<br>T_2: Anthocyanins<br>T_3: Anthocyanins and xanthophylls (lutein + zeaxanthin)	T_1: 8.0<br>T_2: 60<br>T_3: 68	120<br>240	*Systolic*				
						**T_1: Group X**<br>B: 115.0 ± 14.0<br>A120: 115.0 ± 17.0<br>A240: 113.0 ± 13.0	 **0.0**<br>**-2.00**<br>(NS)	**T_2: Group A**<br>B: 118.0 ± 17.0<br>A120: 118.0 ± 17.0<br>A240: 120.0 ± 16.0	 **0.0**<br>**+2.00**<br>(NS)	**Group A *vs* X**<br>**0.0**<br>**+4.00**<br>(NS)
								**T_3: Group A+X**<br>B: 121.0 ± 12.0<br>A120: 118.0 ± 15.0<br>A240: 120.0 ± 12.0	 **-3.0**<br>**-1.0**<br>(NS)	**Group A+X *vs* X**<br>**-3.0**<br>**+1.0**<br>(NS)
										**Group A+X *vs* A**<br>-3.0<br>-3.0<br>(NS)
						Diastolic				
						**T_1: Group X**<br>B: 74.0 ± 10.0<br>A120: 74.0 ± 10.0<br>A240: 74.0 ± 9.0	 **0.0**<br>**0.0**<br>(NS)	**T_2: Group A**<br>B: 76.0 ± 11.0<br>A120: 75.0 ± 9.0<br>A240: 76.0 ± 11.0	 **-1.0**<br>**0.0**<br>(NS)	**Group A *vs* X**<br>**-1.0**<br>**0.0**<br>(NS)
								**T_2: Group A+X**<br>B: 79.0 ± 9.0<br>A120: 78.0 ± 9.0<br>A240: 76.0 ± 10	 **-1.0**<br>**-3.0**<br>(NS)	**Group A+X *vs* C**<br>**0.00**<br>**-3.0**<br>(NS)
										**Group A+X *vs* X**<br>+4.0<br>+2.0<br>(NS)
**Trius-Soler et al., 2021 [32]**	Healthy	Beer beverage with and without alcohol	Prenylflavonoids	T_1: 0.359<br>T_2: 0.259	180	*Systolic*				
								**T_1: Group AB**		**Group AB *vs* C**

		Control (*14*)<br>T1: Group AB (*16*)<br>T2: Group NAB (*7*)				B: 121.0 ± 15.0<br>A: NI	NI<br>(NS)	B: 120.0 ± 14.0<br>A:NI	NI<br>(NS)	**-2.0**<br>(NS)
								**T2: Group NAB**<br>B: 120.0 ± 16.0<br>A: NI	NI<br>(NS)	**Group NAB *vs* C**<br>**-11.0**<br>(NS)
										**Group NAB *vs* AB**<br>**-9.0**<br>(NS)
						Diastolic				
						B: 74.0 ± 13.0<br>A: NI	NI<br>(NS)	**T1: Group AB**<br>B: 73.0 ± 6.0<br>A: NI	NI<br>(NS)	**Group AB *vs* C**<br>**+1.0**<br>(NS)
								**T2: Group NAB**<br>B: 74.0 ± 6.0<br>A: NI	NI<br>(NS)	**Group NAB *vs* C**<br>**-6.0**<br>(NS)
										**Group NAB *vs* AB**<br>-7.0<br>(NS)
**Wang-Polagruto et al., 2006 [25]**	Dyslipidaemia (high cholesterol)	Flavanol cocoa beverage<br><br>T1: Low flavanol (*16*)<br>T2: High flavanol (*16*)	Mix flavanols	T1: 43<br>T2: 446	42	***Systolic***				
						**T1: Low flavanol**<br>B: 140.0 ± 40.0<br>A: 127.0 ± 32.0	**-13.0**<br>(<0.050)	**T2: High flavanol**<br>B: 125.0 ± 32.0<br>A: 128.0 ± 28.0	**+3.0**<br>(<0.050)	**+16.0**<br>(NS)
						Diastolic				
						**T1: Low flavanol**<br>B: 76.0 ± 8.0<br>A: 71.0 ± 12.0	**-5.0**<br>(<0.05)	**T2: High flavanol**<br>B: 64.0 ± 12.0<br>A: 63.0 ± 12.0	**-1.0**<br>(<0.05)	**+4.0**<br>(NS)
**Naissides et al., 2006a [26]**	Dyslipidaemia (high cholesterol)	Control (400 mL water) (*16*)	Mix (poly)phenols	1000	42	***Systolic***				
		T1: Group DRW (400 mL) (*15*)				B: 110.7 ± 15.0<br>A: 113.3 ± 13.0	**+2.6**<br>(NS)	**T1: Group DRW**<br>B: 112.5 ± 18.0<br>A: 110.2 ± 12.0	**-2.3**<br>(NS)	**Group DRW *vs* C**<br>**-4.9**<br>(NS)
		T2: Group RW (400 mL) (*14*)						**T2: Group RW**		**Group RW *vs* C**

								B: 116.8 ± 22.0<br>A: 119.5 ± 18.0	**+2.7**<br>(NS)	**+0.10**<br>(NS)
										**Group DRW *vs* RW**<br>**-5.0**<br>(NI)
								Diastolic		
						B: 67.9 ± 7.6<br>A: 70.0 ± 6.7	**+2.1**<br>(NS)	**T_1: Group DRW**<br>B: 70.5 ± 11.0<br>A: 69.6 ± 12.0	**-0.90**<br>(NS)	**Group DRW *vs* C**<br>**-3.0**<br>(NS)
								**T_2: Group RW**<br>B: 70.1 ± 8.5<br>A: 71.1 ± 12	**+1.0**<br>(NS)	**Group RW *vs* C**<br>**-1.1**<br>(NS)
										**Group DRW *vs* RW**<br>**-1.9**<br>(NI)
**Aubertin-Leheudre et al., 2008 [45]**	Obese	Soybeans capsules (17.5 mg)<br>Placebo (*18*)<br>Treated (*21*)	Mix isoflavones (daidzein, glycitein, and genistein)	70	180			*Systolic*		
						B: 126.4 ± 10.5<br>A: 128.9 ± 11.7	**+2.5**<br>(NI)	B: 125.1 ± 15.0<br>A: 121.9 ± 16.5	**-3.2**<br>(NI)	**-5.7**<br>(NS)
								Diastolic		
						B: 77.6 ± 6.3<br>A: 78.0 ± 7.1	**+0.40**<br>(NI)	B: 79.0 ± 8.7<br>A: 76.7 ± 8.8	**-2.3**<br>(NI)	**-2.7**<br>(NS)
**D`Anna et al., 2014 [31]**	Diagnosis of metabolic syndrome	Capsules (30 mg of cocoa (poly)phenols, 80 mg of soy isoflavones, and 2 g of myo-inositol)<br>Placebo (*21*)<br>Treated (*22*)	Cocoa (poly)phenols and soy isoflavones	110	180			*Systolic*		
						B: 132.6 ± 20.0<br>A: 121.6 ± 9.8	**-11.0**<br>(NS)	B: 123.6 ± 12.0<br>A: 124.0 ± 9.1	**+0.40**<br>(NS)	**+11**<br>(NI)
								Diastolic		
						B: 81.5 ± 11.9<br>A: 70.0 ± 6.3	**-11.5**<br>(NI)	B: 80.0 ± 8.0<br>A: 73.6 ± 11.2	**-6.4**<br>(NS)	**+5.1**<br>(NI)
**Johnson et al., 2015 [29]**	Pre- and Stage 1-Hypertension	Blueberry powder (22 g)<br>Placebo (20)<br>Treated (20)	Mix (poly)phenols (845 mg of phenolic and 46 9mg of anthocyanins)	845	28<br>56			*Systolic*		
						B: 138.0 ± 15.0<br>A28: 136 ± 15.0<br>A56: 139 ± 15.0	**-2.0**<br>**+1.0**<br>(NS)	B: 138.0 ± 14.0<br>A28: 136.0 ± 15.0<br>A56: 131.0 ± 17.0	**-2.0**<br>**-7.0**<br>(<0.05)	**0.0**<br>**-8.0**<br>(<0.01)
								Diastolic		
						B: 78.0 ± 8.0	**0.0**	B: 80.0 ± 7.0	**-3.0**	**-3.0**

	A28: 78.0 ± 11.0	**+2.0**	A28: 77.0 ± 10.0	**-5.0**	**-7.0**
	A56: 80.0 ± 8.0	(NI)	A56: 75.0 ± 9.0	(<0.010)	(<0.010)

Data are presented as the mean ± SD unless otherwise stated; B: Before intervention; A: After intervention; NS: Not significant: NI: Not indicated; C: Control; T: Treated; P: Placebo; NA: Alcoholic beer; NAB: Dealcoholized beer; DRW: Dealcoholized red wine; RW: Red wine; $^{\delta}$: geometric mean [95% CI]; **numbers in bold** are the changes estimated as the mean difference: (A) after – (B) before (intra-group) or treated – control or placebo or comparator (between groups).

Table S9. Changes in inflammatory and endothelial function biomarkers as reported in human RCTs (parallel or crossover design) looking at the chronic effects of (poly)phenol-containing products in postmenopausal women, without hormone replacement therapy.

References	Health status	Treatment Groups (*N* participants per group)	(Poly)phenols	Dose (poly)phenols (mg /d)	Duration (d)	Total circulating biomarker: Control/Placebo/ Comparator	Δ (A – B) (*p*-value)	Treatment	Δ (A – B) (*p*-value)	Δ (Treatment – Control/Placebo) (*p*-value)
						**TNF-α, IL-6, CRP**				
**Johnson et al., 2017 [30]**	Pre- and Stage 1-Hypertension	Blueberry powder (22 g)	Mix (poly)phenols (845 mg of phenolic and 469 mg of anthocyanins)	1314	28	**TNF-α (pg/mL; serum)**				
					56	B: 5.1 ± 1.3	**-2.0**	B: 6.7 ± 3.0	**-2.0**	**+0.11**
		Placebo (*20*)				A28: 3.0 ± 0.89	**-1.0**	A28: 4.8 ± 3.0	**-2.0**	**-0.17**
		Treated (*20*)				A56: 3.7 ± 0.67	(<0.050)	A56: 5.1 ± 2.8	(<0.050)	(0.020)
						**CRP (mg/mL; serum)** Ŏ				
						B: 2.7 ± 3.6	**-0.05**	B: 2.5 ± 3.9	**-0.05**	**0.0**
						A28: 2.6 ± 3.9	**-0.40**	A28: 2.4 ± 4.5	**-0.19**	**+0.21**
						A56: 2.3 ± 5.4	(NS)	A56: 2.3 ± 4.8	(NS)	(NS)
**Zern et al., 2005 [40]**	Healthy	Lyophilized grape powder	Mix grape (poly)phenols (anthocyanins, quercetin, myricetin, kaempferol and resveratrol)	~210	28	**TNF-α (ng/L; plasma)**				
		Placebo (*20*)				B: NI	NI	B: NI	NI	NI
		Treated (*20*)				A: 2.5 ± 1.8	(NI)	A: 2.2 ± 2.0	(NI)	(<0.050)
						**IL-6 (pg/L; plasma)**				
						B: NI	NI	B: NI	NI	NI
						A: 2.3 ± 1.2	(NI)	A: 2.2 ± 1.3	(NI)	(NS)
						**CRP (mg/L; plasma)**				
						B: NI	NI	B: NI	NI	NI
						A: 4.9 ± 7.7	(NI)	A: 4.6 ± 8.9	(NI)	(NS)
**Curtis et al., 2009 [39]**	Healthy	Elderberry extracts capsules (125 mg)	Mix anthocyanins (mostly cyanidin-3-glucoside) per capsule	500	84	**TNF-α (ng/L; plasma)**				
						B: 15.0 ± 9.3	**-2.0**	B: 15.0 ± 11.0	**-5.0**	**-3.0**
		Placebo (*26*)				A: 13.0 ± 9.2	(NS)	A: 11.0 ± 5.5	(NS)	(NS)
		Treated (*26*)				**IL-6 (ng/L; plasma)**				
						B: 1.0 ± 1.4	**-0.10**	B: 1.0 ± 0.9	**0.0**	**+0.10**
						A: 0.90 ± 0.90	(NS)	A: 1.0 ± 0.6	(NS)	(NS)
						**CRP (mg/L; plasma)**				
						B: 0.90 ± 0.90	**0.0**	B: 1.3 ± 1.0	**0.0**	**0.0**
						A: 0.90 ± 0.70	(NS)	A: 1.3 ± 1.1	(NS)	(NS)
**Filip et al., 2015 [28]**	Osteopenia (mix cholesterol levels)	Olive leaf extract	Mix (poly)phenols (>40% oleuropein)	>100 oleuropein	365	**IL-6 (pg/mL; serum)**				
		Placebo (400 mg Ca capsules) (*21*)				B: 1.8 ± 1.4	**+0.11**	B: 2.0 ± 2.1	**0.0**	**-0.11**
						A: 1.9 ± 1.2	(NS)	A: 2.0 ± 2.2	(NS)	(NS)
						**CRP (mg/L; plasma)**				

		Treated (400 mg Ca + 250 mg olive extract capsules) (*27*)				B: 3.2 ± 1.6	**-0.77**	B: 2.8 ± 1.2	**+0.34**	**+1.0**
						A: 2.5 ± 1.9	(NS)	A: 3.2 ± 2.3	(NS)	(NS)
**Chai et al., 2012 [41]**	Healthy	Dried plum or dried apple	Mix (poly)phenols	NI	90	**CRP (mg/dL; serum)**				
		T_1: Group DP (*55*)			180	**T_1: Group DP**		**T_2: Group DA**		**Group DA *vs* DP**
		T_2: Group DA (*45*)			365	B: 1.8 ± 5.6	**-0.30**	B: 2.1 ± 3.5	**0.0**	**+0.30**
						A90: 1.5 ± 3.0	**-0.20**	A90: 2.1 ± 2.9	**-0.50**	**-0.30**
						A180: 1.6 ± 2.9	**-0.40**	A180: 1.6 ± 2.7	**-0.70**	**-0.30**
						A365: 1.4 ± 5.7	(0.019)	A365: 1.4 ± 4.8	(0.019)	(NI)
**Sathyapalan et al., 2018 [37]**	Healthy	Snack bars (soy protein free isoflavones or with isoflavones)	Mix isoflavones	66	180	**CRP (mg/L; NI)**				
						B: 2.7 ± 4.5	-0.50	B: 1.7 ± 1.6	-0.96	-0.46
						A: 2.1 ± 2.3	(NI)	A: 0.69 ± 0.92	(NI)	(NS)
		Placebo (*60*)								
		Treated (*60*)								
**Estévez-Santiago et al., 2019 [44]**	Healthy	Capsules	T_1: Xanthophylls (lutein + zeaxanthin)	T_1: 8.0	120	**IL-6 (pg/ml; plasma)**				
		T_1: Group X *(26)*	T_2: Anthocyanins	T_2: 60	240	**T_1: Group X**		**T_2: Group A**		**Group A *vs* X**
		T_2: Group A *(23)*	T_3: Anthocyanins and xanthophylls (lutein + zeaxanthin)	T_3: 68		B: 1.0 ± 0.60	**+0.10**	B: 0.80 ± 0.20	**+0.60**	**+0.50**
		T_3: Group A+X *(23)*				A240: 1.1 ± 0.80	(NS)	A240: 1.4 ± 1.9	(NS)	(NS)
								**T_3: Group A+X**		**Group A+X *vs* X**
								B: 1.1 ± 0.50	**+0.10**	**0.0**
								A240: 1.2 ± 0.90	(NS)	(NS)
										**Group A+X *vs* A**
										**-0.50**
										(NS)
						**CRP (mg/L; serum)**				
						**T_1: Group X**		**T_2: Group A**		**Group A *vs* X**
						B: 1.5 ± 1.8	**+0.30**	B: 1.2 ± 0.89	**+0.60**	**+0.30**
						A120: 1.8 ± 2.2	**-0.10**	A120: 1.8 ± 1.9	**+0.10**	**+0.20**
						A240: 1.4 ± 1.2	(NS)	A240: 1.3 ± 1.2	(NS)	(NS)
								**T_3: Group A+X**		**Group A+X *vs* X**
								B: 1.4 ± 1.3	**+0.10**	**-0.20**
								A120: 1.5 ± 2.0	**-0.20**	**-0.10**
								A240: 1.2 ± 1.5	(NS)	(NS)
										**Group A+X *vs* A**
										**-0.50**
										**-0.30**
										(NS)

**ADIPONECTIN**										
**Wu et al., 2012 [38]**	Healthy	Green tea extract capsules<br>Placebo *(32)*<br>T_1: Group G400 *(37)*<br>T_2: Group G800 *(34)*	Mix (poly)phenols (mostly EGCG plus EC, EGC, ECG, and GCG)	T_1: 400<br>T_2: 800	60	**(µg/mL; serum)**<br>B: 17.1 [14.0, 20.0] $^{\delta}$<br>A: 18.0 [15.0, 21.0] $^{\delta}$	**+0.90**<br>(NS)	**T_1: Group G400**<br>B: 16.4 [14.1, 19.1] $^{\delta}$<br>A: 16.3 [14.0, 19.0] $^{\delta}$<br>**T_2: Group G800**<br>B: 17.0 [15.0, 20.0] $^{\delta}$<br>A: 16.7 [14.0, 20.0] $^{\delta}$	**G400**<br>**-0.10**<br>(NS)<br>**G800**<br>**-0.30**<br>(NS)	**Group G400 *vs* p**<br>**-1.0**<br>(NS)<br>**Group G800 *vs* P**<br>**-1.0**<br>(NS)<br>**Groups *vs* P**<br>NI<br>(NS)<br>**Group G400 *vs* G800**<br>**-0.20**<br>(NS)
**D'Anna et al., 2014 [31]**	Diagnosis of metabolic syndrome	Capsules (30 mg of cocoa (poly)phenols, 80 mg of soy isoflavones, and 2 g of myo-inositol)<br>Placebo (*21*)<br>Treated (*22*)	Cocoa (poly)phenols and of soy isoflavones	110	180	**(µg/mL; serum)**<br>B: 22.0 ± 5.0<br>A: 16.0 ± 7.0	**-6.0**<br>(0.001)	B: 18.0 ± 6.0<br>A: 17.0 ± 4.0	**-1.0**<br>(NS)	**+5.0**<br>(<0.001)
**Dostal et al., 2016 [33]**	Overweight/ obese	Capsules (1315 mg of catechins)<br>Placebo *(120)*<br>Treated *(117)*	Catechins	1315	365	**(µg/mL; plasma)**<br>B: 6.8 [6.1, 7.6] $^{\delta}$<br>A: 6.6 [5.9, 7.4] $^{\delta}$	+0.11<br>(0.14)	B: 6.4 [5.7, 7.2] $^{\delta}$<br>A: 6.6 [5.9, 7.4] $^{\delta}$	+0.72<br>(0.14)	**+0.61**<br>(NS)
**ENDOTHELIAL FUNCTION**										
**Estévez-Santiago et al., 2019 [44]**	Healthy	Capsules<br>T_1: Group X *(26)*<br>T_2: Group A *(23)*<br>T_3: Group A+X *(23)*	T_1: Xanthophylls (lutein + zeaxanthin)<br>T_2: Anthocyanins<br>T_3: Anthocyanins and xanthophylls (lutein + zeaxanthin)	T_1: 8.0<br>T_2: 60<br>T_3: 68	120<br>240	**sVCAM-1 (ng/mL; plasma)**<br>**T_1: Group X**<br>B: 174.0 ± 62.0<br>A240: 160.0 ± 66.0	**-14.0**<br>(NS)	**T_2: Group A**<br>B: 185.0 ± 76.0<br>A240: 166.0 ± 54.0<br>**T_3: Group A+X**<br>B: 152.0 ± 52.0<br>A240: 149.0 ± 83.0	**-19.0**<br>(NS)<br>**-3.0**<br>(NS)	**Group A *vs* X**<br>**-5.0**<br>(NS)<br>**Group A+X *vs* X**<br>**+11.0**<br>(NS)<br>**Group A+X *vs* A**<br>**+16.0**<br>(NS)
						**sICAM-1 (ng/mL; plasma)**<br>**T_1: Group X**		**T_2: Group A**		**Group X *vs* A**

						B: 344.0 ± 179.0<br>A240: 320.0 ± 132.0	**-24.0**<br>(NS)	B: 381.0 ± 208.0<br>A240: 351.0 ± 122.0	**-30.0**<br>(NS)	**-6.0**<br>(NS)
								**T_3: Group A+X**<br>B: 346.0 ± 192.0<br>A240: 345.0 ± 106.0	**-1.0**<br>(NS)	**Group A+X *vs* X**<br>**+23.0**<br>(NS)<br><br>**Group A+X *vs* A**<br>**+29.0**<br>(NS)
**Wang-Polagruto et al., 2006 [25]**	Dyslipidaemia (high cholesterol)	Flavanol cocoa beverage<br>T_1: Low flavanol (*16*)<br>T_2: High flavanol (*16*)	Mix flavanols	T_1: 43<br>T_2: 446	42	**sVCAM-1 (ng/mL; plasma)**<br>**T_1: Low flavanol**<br>B: 399.0 ± 138.0<br>A: 434.0 ± 186.0	**+35.0**<br>(NS)	**T_2: High flavanol**<br>B: 632.0 ± 148.0<br>A: 554.0 ± 107.0	**-78.0**<br>(0.009)	**-113.0**<br>(0.010)
						**sICAM-1 (ng/mL; plasma)**<br>**T_1: Low flavanol**<br>B: 166.0 ± 48.0<br>A: 162.0 ± 50.0	**-3.0**<br>(NS)	**T_2: High flavanol**<br>B: 183.0 ± 32.0<br>A: 183.0 ± 44.0	**-0.20**<br>(NS)	**+3.0**<br>(NS)
						**sP-Selectin (ng/mL; plasma)**<br>**T_1: Low flavanol**<br>B: 31.0 ± 17.0<br>A: 32.0 ± 18.0	**+0.90**<br>(NS)	**T_2: High flavanol**<br>B: 28.0 ± 9.6<br>A: 32.0 ± 14.0	**+4.0**<br>(NS)	**+3.0**<br>(NS)
						**sE-Selectin (ng/mL; plasma)**<br>**T_1: Low flavanol**<br>B: 45.0 ± 17.0<br>A: 47.0 ± 21.0	**+2.0**<br>(NS)	**T_2: High flavanol**<br>B: 45.0 ± 19.0<br>A: 42.0 ± 17.0	**-3.0**<br>(NS)	**-5.0**<br>(NS)

Data are presented as the mean ± SD unless otherwise stated; $^{\delta}$: geometric mean [95% CI]; B: Before intervention; A: After intervention; NS: Not significant; NI: Not indicated; C: Control; P: Placebo; T: Treated; EGCG: Epigallocatechin gallate; EC: Epicatechin; EGC: Epigallocatechin; ECG: Epicatechin gallate; GCG: Gallocatechin gallate; DP: Dried plum; DA: Dried apple; Ca: Calcium; **numbers in bold** are the changes estimated as the mean difference: (A) after – (B) before (intra-group) or treated – control or placebo or comparator (between groups); $^{\breve{O}}$: The same data appear in the article by Johnson et al., 2015.

Table S10. Changes in oxidative stress biomarkers as reported in human RCTs (parallel or crossover design) looking at the chronic effects of (poly)phenol-containing products in postmenopausal women, without hormone replacement therapy.

References	Health status	Treatment Groups (*N* participants per group)	(Poly)phenols	Dose (poly)phenols (mg /d)	Duration (d)	Total circulating biomarker: Control/Placebo/ Comparator	Δ (A – B) (*p*-value)	Treatment	Δ (A – B) (*p*-value)	Δ (Treatment – Control/Placebo) (*p*-value)
CHEMICAL BIOMARKERS										
**Johnson et al., 2017 [30]**	Pre- and Stage 1-Hypertension	Blueberry powder (22 g)<br><br>Placebo (*20*)<br>Treated (*20*)	Mix (poly)phenols (845 mg of phenolic and 469 mg of anthocyanins)	845	28<br>56	**Ox-LDL (ng/mL; serum)**<br>B: 424.0 ± 37.0<br>A28: 319.4 ± 60.0<br>A56: 408.8 ± 133.0	 **-104.6**<br>**-15.0**<br>(28d)<br>(<0.050)	 B: 383.0 ± 24.0<br>A28: 337.9 ± 38.0<br>A56: 400.4 ± 21.0	 **-45.1**<br>**+17.4**<br>(28d)<br>(<0.050)	 **+59.5**<br>**+32.4**<br>(NS)
						**Isoprostanes (pg/mL; serum)**<br>B: 15.1 ± 14.0<br>A28: 9.9 ± 5.8<br>A56: 11.9 ± 7.5	 **-5.2**<br>**-3.2**<br>(28d)<br>(<0.050)	 B: 18.6 ± 8.4<br>A28: 15.9 ± 8.3<br>A56: 11.8 ± 4.6	 **-2.7**<br>**-6.8**<br>(56d)<br>(<0.050)	 **+2.5**<br>**-3.6**<br>(NS)
						**8-OHdG (ng/mL; plasma)**<br>B: 0.31 ± 0.04<br>A28: 0.35 ± 0.92<br>A56: 0.35 ± 0.04	 **+0.04**<br>**+0.04**<br>(NS)	 B: 0.32 ± 0.04<br>A28: 0.28 ± 0.04<br>A56: 0.32 ± 0.06	 **-0.04**<br>**0.0**<br>(NS)	 **-0.08**<br>**-0.04**<br>(28d)<br>(0.008)
						**TBARS (µM; serum)**<br>B: 2.0 ± 0.60<br>A28: 2.5 ± 1.7<br>A56: 1.9 ± 0.40	 **+0.50**<br>**-0.10**<br>(NS)	 B: 1.9 ± 0.50<br>A28: 2.7 ± 1.5<br>A56: 2.2 ± 0.80	 **+0.80**<br>**+0.30**<br>(NS)	 **+0.30**<br>**+0.40**<br>(NS)
**Zern et al., 2005 [40]**	Healthy	Lyophilized grape powder<br><br>Placebo (*20*)<br>Treated (*20*)	Mix grape (poly)phenols (anthocyanins, quercetin, myricetin, kaempferol and resveratrol)	~210	28	**Isoprostanes (ng/mg creatinine urine)**<br>B: NI<br>A: 1.1 ± 0.80	 NI<br>(NI)	 B: NI<br>A: 1.0 ± 0.95	 NI<br>(NI)	 NI<br>(S)
**Chai et al., 2012 [41]**	Healthy	Dried plum or dried apple<br>T_1: Group DP (*55*)	Mix (poly)phenols	NI	90<br>180	**LPO (µmol/L; serum)**<br>**Group DP**		 **Group DA**		 **Group DA *vs* DP**

		T$_2$: Group DA (*45*)			365	B: 52.4 ± 43	**-12.4**	B: 58.9 ± 43.0	**-12.2**	**+0.20**
						A90: 40.0 ± 65	**+7.9**	A90: 46.7 ± 56.0	**+0.20**	**-7.7**
						A180: 60.3 ± 25	**-19.8**	A180: 59.1 ± 27.0	**-19.7**	**+0.10**
						A365: 32.6 ± 29	(0.001)	A365: 39.2 ± 27.0	(0.022)	(NS)
**ANTIOXIDANTS ENZYMES**										
**Johnson et al., 2017 [30]**	Pre- and Stage 1-Hypertension	Blueberry powder (22 g)	Mix (poly)phenols (845 mg of phenolic and 469 mg of anthocyanins)	1314	28	**SOD (U/mL; serum)**^{δ}				
					356	B: 0.23 ± 0.22	**+0.17**	B: 0.21 ± 0.27	**+0.15**	**-0.02**
		Placebo (*20*)				A28: 0.40 ± 0.27	**+0.26**	A28: 0.36 ± 0.49	**+0.29**	**+0.03**
		Treated (*20*)				A56: 0.49 ± 0.67	(<0.001)	A56: 0.50 ± 0.98	(<0.001)	(NS)
						**GSR (nmol/min/mL; plasma)**				
						B: 8.8 ± 4.0	**+5.3**	B: 8.9 ± 4.4	**+5.2**	**-0.1**
						A28: 14.1 ± 5.3	**+8.1**	A28: 14.1 ± 4.7	**+6.7**	**-1.4**
						A56: 16.8 ± 4.0	(<0.050)	A56: 15.6 ± 3.9	(<0.05)	(NS)
						**GPx (nmol/min/mL; plasma)**				
						B: 29.4 ± 30.0	**+58.8**	B: 29.8 ± 22	**+61.9**	**+3.1**
						A28: 88.2 ± 25.0	**+19.3**	A28: 91.7 ± 23	**+34.4**	**+15.1**
						A56: 48.7 ± 25.0	(<0.05)	A56: 64.2 ± 37	(<0.05)	(NS)

Data are presented as the mean ± SD; B: Before intervention; A: After intervention; NS: not significant; NI: not indicated; C: Control; T: Treated; P: Placebo; DP: Dried plum; DA: Dried apple; **numbers in bold** are the changes estimated as the mean difference: (A) after – (B) before (intra-group) or treated – control or placebo or comparator (between groups). Ox-LDL: oxidized LDL; 8-OHGdG: 8-hydroxy-guanosine TBARS: thiobarbituric acid reactive substances; LPO: lipid hydroperoxide; SOD; superoxide dismutase; GSR: glutathione reductase; GPX: glutathione peroxidase; ^{δ}: The same data appear in the article by Johnson et al., 2015.
